# Genetic divergence, population differentiation and phylogeography of the cicada *Subpsaltria yangi* based on molecular and acoustic data: an example of the early stage of speciation?

**DOI:** 10.1186/s12862-018-1317-8

**Published:** 2019-01-08

**Authors:** Yunxiang Liu, Christopher H. Dietrich, Cong Wei

**Affiliations:** 1 State Key Laboratory of Crop Stress Biology for Arid Areas, and Key Laboratory of Plant Protection Resources and Pest Management, Ministry of Education, College of Plant Protection, Northwest A&F University, Yangling, 712100 Shaanxi China; 2 Illinois Natural History Survey, Prairie Research Institute, University of Illinois, Champaign, IL 61820 USA

**Keywords:** Acoustic signals, Allopatric speciation, Cicadidae, Geographical isolation, Northern China, Population differentiation

## Abstract

**Background:**

Geographical isolation combined with historical climatic fluctuations have been identified as two major factors that contribute to the formation of new species. On the other hand, biotic factors such as competition and predation are also able to drive the evolution and diversification of organisms. To determine whether geographical barriers contributed to population divergence or speciation in the rare endemic cicada *Subpsaltria yangi* the population differentiation, genetic structure and phylogeography of the species were investigated in the Loess Plateau and adjacent areas of northwestern China by analysing mitochondrial and nuclear DNA and comparing the calling song structure of 161 male individuals.

**Results:**

The results reveal a low level of genetic differentiation and relatively simple phylogeographic structure for this species, but two independent clades corresponding to geographically isolated populations were recognised. Genetic and geographical distances were significantly correlated among lineages. Results of divergence-time estimation are consistent with a scenario of isolation due to glacial refugia and interglacial climate oscillation in northwestern China. Significant genetic divergence was found between the population occurring in the Helan Mountains and other populations, and recent population expansion has occurred in the Helan Mountains and/or adjacent areas. This population is also significantly different in calling song structure from other populations.

**Conclusions:**

Geographical barriers (i.e., the deserts and semi-deserts surrounding the Helan Mountains), possibly coupled with related ecological differences, may have driven population divergence and allopatric speciation. This provides a possible example of incipient speciation in Cicadidae, improves understanding of population differentiation, acoustic signal diversification and phylogeographic relationships of this rare cicada species of conservation concern, and informs future studies on population differentiation, speciation and phylogeography of other insects with a high degree of endemism in the Helan Mountains and adjacent areas.

**Electronic supplementary material:**

The online version of this article (10.1186/s12862-018-1317-8) contains supplementary material, which is available to authorized users.

## Background

Geographic isolation is essential to most speciation events, because biogeographic barriers (due to distance, water bodies, mountains, deserts, etc.) separate populations, impede gene flow, and drive genetic differentiation, which may lead to allopatric speciation [1–3]. The mosaic of high mountains in East Asia forms a complex terrain imposing geographical barriers hindering movement of many animal species [1, 4]. The uplift of the Qinghai-Tibet Plateau (about 1.7–3.6 million years ago (Ma)), in particular, had a profound impact on the surrounding geography and environment, generating sources and reservoirs of genetic and species diversity [5, 6]. Previous studies of East Asian insects confirmed that the complex local topography and geographic isolation had a marked effect on the distribution and demography of many insects, e.g., *Bactrocera cucurbitae* (Diptera: Tephritidae) [7], *Apocheima cinerarius* (Lepidoptera: Geometridae) [8], *Panesthia* cockroaches (Blattaria: Blaberidae) [9], and *Halyomorpha halys* (Hemiptera: Pentatomidae) [10]. During the past three million years, global climate has fluctuated greatly, which resulted in the recent major ice age [11], and substantial changes in the distributions of many living organisms, expressed differently in temperate and tropical zones [12, 13]. During the ice age, mountainous areas are thought to have harboured many refugial populations, which led to the formation of new lineages/taxa and contributed to higher genetic diversity [14, 15]. Such changes include fragmentation of previously contiguous populations which, in animals, may result in both genetic and behavioral divergence. In particular, acoustic signals function in species recognition and mate choice in a wide range of taxa. It has been proposed that divergence in acoustic signals often plays an important part in speciation, and study of such signals has been used to discover examples of incipient or cryptic speciation [16, 17].

The increasing desertification of northern China caused by the uplift of the QTP effectively changed the biodiversity of this area [18, 19]. The Helan (HL) Mountains and adjacent areas of northern China, which were first formed during the Mesozoic between 205 and 135 Ma and reach a maximum elevation of 3556 m, may be an ideal location to investigate the effects of geographical isolation on speciation [20, 21]. These mountains are considered an important area for biodiversity conservation in China because they provide suitable habitats and environmental conditions for many highly endemic species [22, 23]. The mountains are surrounded by deserts and semi*-*deserts which may serve as geographical barriers to species with limited dispersal ability [24], and the isolated peaks may be equivalent to islands biogeographically [25, 26]. The patchwork of mountains, deserts and semi*-*deserts in this region may promote the differentiation of populations and increase the likelihood of speciation.

The large body size and low dispersal ability of cicadas has led to their being used as model organisms for phylogeographic and speciation studies in different regions of the world including New Zealand [27, 28], South Africa [29], North America [30] and the Mediterranean area [31]. The cicada *Subpsaltria yangi* Chen is the only known species of the tribe Tibicininii in China. The species was previously placed in the subfamily Tettigadinae [32, 33] but was removed recently to the subfamily Tibicininae [34]. This rare endemic species distributed in the Loess Plateau and adjacent areas of northwestern China [35] is unusual in that the males possess a well-developed stridulatory mechanism in addition to the timbals, and in that the females also possess the same stridulatory organs as males [32, 33]. The species was, until recently, considered to be extinct, because it had not been found in the field since 1980s and was only known from museum specimens collected from the center of Shaanxi Province. Nevertheless, *S. yangi* was rediscovered in June of 2011 during a survey of the insect fauna of the Helan Mountains which is surrounded by deserts and semi-desert. This provided an opportunity to study this little-known species, and led to the discovery of the positive phonotaxis and acoustical sexual mimicry in males [35–37]. During our field investigations of *S. yangi* since 2011, a few more populations were discovered from the Loess Plateau in Shaanxi, Shanxi and Gansu provinces, which were all attracted and sampled using our special acoustic playback method [35]. Interestingly, the habitats inhabited by the species in the Helan Mountains and the Loess Plateau are greatly divergent. Populations of *S. yangi* occurring in the Helan Mountains mainly feeds on *Ephedra lepidosperma* C.Y. Cheng (Ephedraceae), a shrub endemic to the Helan Mountains, but populations distributed in the Loess Plateau mainly feed on *Ziziphus jujuba* Mill. var. *spinosa* (Bunge) Hu ex H. F. Chow (Rhamnaceae) [35–37]. The differences in habitats and diets suggest that niche expansion and significant genetic divergence may have occurred among populations of this cicada species.

To determine whether geographical barriers are important factors driving population divergence or allopatric speciation of *S. yangi*, we explored the pattern of genetic divergence among populations of this species using phylogeographic analyses based on mitochondrial and nuclear gene sequences. The genetic distance and divergence times were estimated to assess the potential influence of historical climatic fluctuations on population differentiation and niche expansion. The calling song structures of males of four representative populations were also investigated and compared to further clarify the extent of population differentiation or speciation.

## Materials and methods

### Species and sampling

**Fig. 1:**
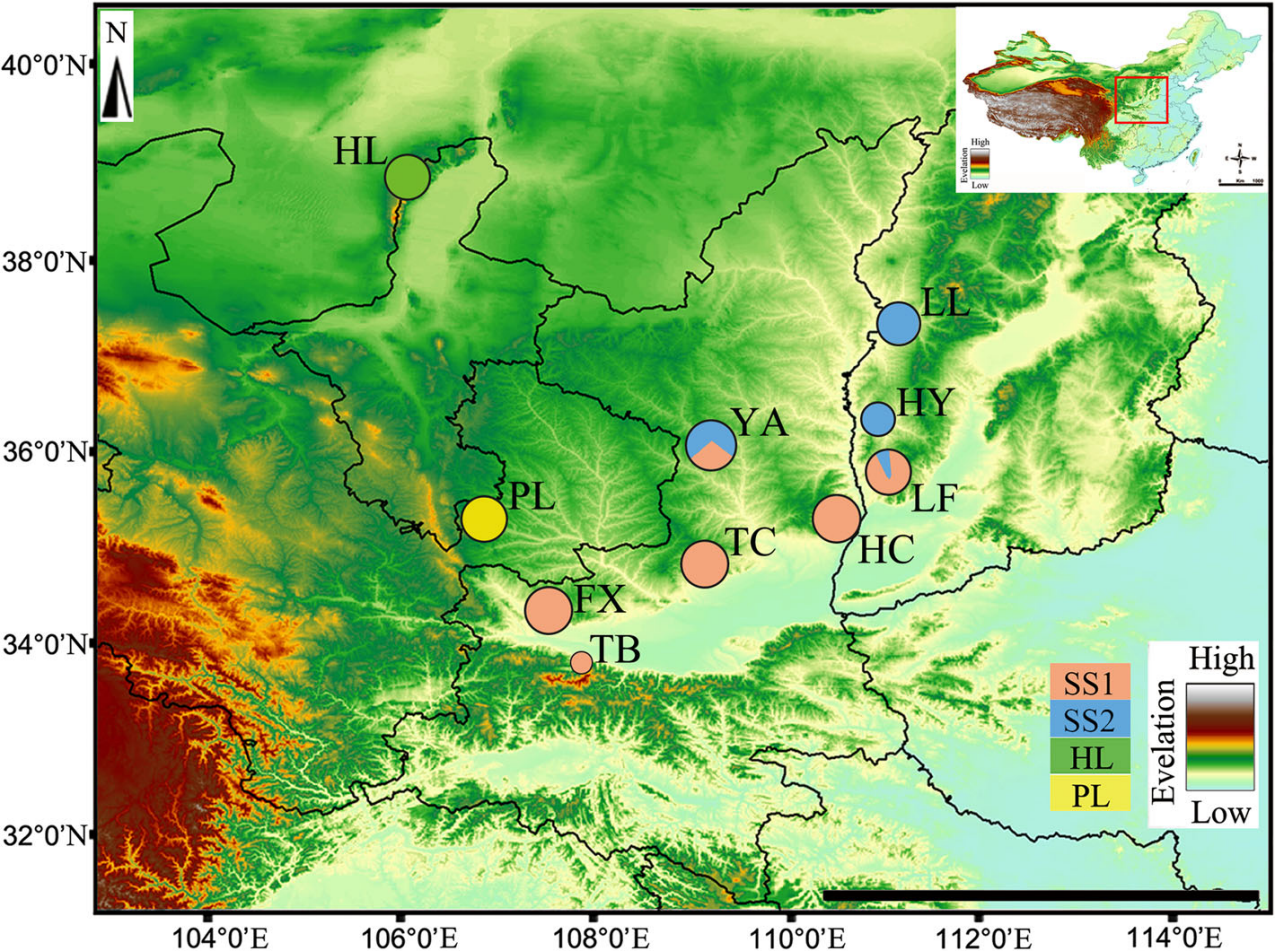
The geographic distribution of *S. yangi* species sampled in this study. Detailed information on the locations population codes are presented in Table 1. Pie charts for each population correspond to the proportion of each genetic cluster in the population. Figure generated in ArcGIS 10 (Environmental Systems Research Institute). Size of circles indicate sample size. SS: Group in Shaanxi and Shanxi province. HL: Population in Ningxia Hui Nationality Autonomous Region. PL: Population in Gansu province

Samples were collected throughout the formerly documented range and potential range of *S. yangi*, including Shaanxi Province, Shanxi Province, Gansu Province, and Ningxia Hui Autonomous Region. In total, 161 specimens, representing ten natural populations, were collected from 2013 to 2017. Sweep netting and acoustical trap sampling were used to collected individuals from different drought-tolerant dwarf shrubs or herbaceous plants, which generally do not exceed 1 m in height in the dry habitats sampled. Sampling site and sample-size information are summarized in Table 1 and depicted in Fig. 1. Tissue samples were frozen in individually labeled containers in 100% EtOH. All vouchers are deposited in the Entomological Museum of Northwest A&F University (NWAFU), Yangling, China.

**Table 1:** Occurrence data for the 10 populations of *S. yangi* used for molecular analyses in this study

Population	Collection location	Longitude (E)	Latitude (N)	Collection date	Number of individuals
FX	Fengxiang, Shaanxi	107°23′20″	34°31′56″	15/6/2016	20
HC	Hancheng, Shaanxi	109°35′54″	34°54′35″	25/5/2014	18
HL	Helan Mountains, Ningxia	106°51′20″	38°57′47″	5/6/2015	20
LF	Linfen, Shanxi	111°03′40″	36°04′37″	3/6/2015	20
LL	Luliang, Shanxi	111°34′50″	37°39′54″	7/6/2015	18
PL	Pingliang, Gansu	106°39′54″	35°51′00″	20/6/2016	17
TB	Taibai, Shaanxi	107°31′22″	34°02′46″	14/6/2017	2
TC	Tongchuan, Shaanxi	108°34′56″	34°50′36″	14/6/2014	18
YA	Yan’an, Shaanxi	109°28′33″	36°50′50″	29/5/2014	20
YH	Yonghe, Shanxi	111°05′20″	36°58′48″	6/6/2015	10

### DNA extraction, amplification and sequencing

A Biospin Insect Genomic DNA Extraction Kit (Bioer Technology Co., Ltd., Hangzhou, China) was used to extract total genomic DNA from leg muscle following the manufacturer’s instructions. Standard polymerase chain reaction (PCR) methods were used to amplify partial sequences of four mtDNA regions (cytochrome c oxidase subunit I (*COI*), cytochrome c oxidase subunit II (*COII*), cytochrome b (*Cytb*), and ATPase subunits (*A6A8*)) and two nuclear gene segments (internal transcribed spacer 1 (*ITS1*) and the elongation factor-1 alpha gene (*EF-1α*)) from representative samples. The primer pairs used in this study for these six genes are presented in Additional file 1: Table S1. PCR programs were conducted in a total volume of 25 μl using the following thermal cycling conditions: an initial denaturation at 95 °C for 3 min, followed by 30 cycles of 95 °C for 30 s, 44.9–59.3 °C for 45 s (51.4 °C for *COI*, 44.9 °C for *COII*, 54.4 °C for *Cytb*, 47.7 °C for *A6A8*, 59.3 °C for *ITS1* and 54.4 °C for *EF-1α*), and an extension at 72 °C for 60 s, and a final extension step of 72 °C for 7 min. PCR products were visualized on a 1% agarose gel with ethidium bromide staining. PCR products were then purified and sequenced in both directions by Shanghai Sangon Biological Engineering Technology and Service Co., Ltd. All sequences gathered in this study have been deposited in GenBank (Accession Nos.: *COI*: MG279557–MG279685; *COII*: MG592751–MG592882; *Cytb*: MG387233–MG387361; *A6A8*: MG281557–MG281685; *ITS1*: MG592883–MG593021; *EF-1α*: MG387362–MG387402).

### Phylogenetic analysis

The chromatograms of each sequence were proofread and then assembled using SEQMAN PRO (DNAstar, Madison). Each protein*-*coding sequence was translated for confirmation and assignment of codon positions using PREMIER v5.0 (Premier Biosoft International, Palo Alto, CA). Multiple sequence alignment was carried out in CLUSTAL X v1.81 [38], with gappy columns at the beginning and end of the alignment manually deleted with BIOEDIT v7.0.9.0 [39]. To confirm the alignment, PRIMER PREMIER v5 [40] was used to translate the sequences into amino acids. The alignment applied for phylogenetic analyses was measured for its sensitivity to misaligned regions identified with the program GBLOCKS [41], as no repeatable criteria in the manual alignment could be used to determine which regions were divergent or ambiguously aligned by BIOEDIT. DAMBE v5.3.74 [42] was conducted to test the level of substitution saturation of each gene and each codon position of each protein-coding gene. No insertions, deletions, or stop codons were present in the alignment. Base frequency and the number of variable and parsimony informative sites were calculated in MEGA v6 [43]. We tested for homogeneity of base frequencies across taxa for each gene using the chi-square test implemented in PAUP* v4.0b10 [44]. Mean sequence divergences among the major clades were calculated using MEGA v6 under the pairwise Kimura two*-*parameter (K2P) model.

The program PARTITIONFINDER v1.1.1 [45] was used to estimate evolutionary models and the most suitable partitioning strategies for each partition, with different potential groups of 1st, 2nd and 3rd codon positions of protein-coding genes given to the program. Sequence models and alternative partitioning were compared using the Bayesian Information Criterion (BIC). Bayesian analysis (BI) was conducted using MrBayes v3.1.2 [46]. Maximum likelihood (ML) analysis was tested using the program raxmlGUI v1.3, a graphical front-end for RAxML [47]. Under the GTR + I + G model we ran ten times for all maximum likelihood analyses with the “thorough” bootstrap setting, starting from random seeds. This was repeated until the likelihood score and parameter estimates no longer changed. In order to keep the best tree only, trees were initially evaluated under maximum parsimony by stepwise random addition with tree bisection*-*reconnection (TBR) branch swapping on ten replicates. Bootstraps [48] were conducted for the ML analyses using the final parameter settings for 100 pseudo replicates, saving the best tree from ten search replicates per bootstrap replicate. The bootstrap support value (BS) was estimated by analysis with 1000 replicates. We initially analysed the entire dataset (*COI + COII + Cytb + A6A8 + EF-1α + ITS1)*, and performed subsequent analyses on the (*COI + COII + Cytb + A6A8*) and (*EF-1α + ITS1)* datasets, respectively. Sequences from two closely related species, *Okanagana utahensis* and *O. canadensis*, obtained from Sueur et al. [49], were used as outgroup taxa for the phylogenetic analysis.

BI analysis was conducted using MrBayes v3.1.2. The Markov chain Monte Carlo (MCMC) algorithm was run for 2,000,000 generations, with four incrementally heated chains. The analysis involved starting from a random tree and sampling every 100 generations. The average standard deviation of split frequency among runs was lower than 0.01, indicating that the sampling of posterior distribution was adequate. Convergence was examined using Potential Scale Reduction Factor (PSRF) and the average standard deviation of split frequencies. Stationarity was determined using TRACER v1.5 [50] by plotting the log-likelihood values versus generation number. A50% majority-rule consensus tree with the posterior probability values was constructed by summarizing the remaining trees, before discarding the first 25% of the yielded trees as burn-in.

### Population structure and history demography analysis

The numbers of haplotypes (H), the values of haplotype diversity (h) and nucleotide diversity (π) [51] for every sampling site, lineage and population were estimated by DNASP v5.0 [51]. The neighbor-net algorithm with SPLITSTREE v4.6 [52], and a median-joining method performed with default settings in NETWORK v5.0 [53] were used to construct unrooted networks in order to explore the relationships among unique haplotypes. Populations were divided into haplogroups according to preceding analysis. A three-level hierarchical analysis of molecular variance (AMOVA) [54] was performed with genetic variation and fixation indices implemented in ARLEQUIN v3.5 [55] by computing conventional F-statistics from haplotypes with 1000 permutations. To explore the historical population demographics and examine whether the sequences conformed to the expectations of neutrality, Tajima’s D and Fu’s F statistic were computed, and 10,000 coalescent simulations were performed for each statistic to create 95% confidence intervals.

The model-based Bayesian Analysis of Population Structure (BAPS) v6.0 was used to study the genetic structure of *S. yangi* [56]*.* The genetic clusters probabilities (from K = 1 to K = 20) were surveyed with *COI* gene under “mixture analysis” and “spatial clustering of individuals” models [57]. In addition, BAPS selected 10 as the number of interactions used to assess the admixture coefficients for the reference individuals, 200 as the number of reference individuals from each genotype and 1000 as the number of interactions to assess the admixture coefficients for the genotypes.

Pairwise mismatch distribution analyses were carried on to detect evidence of past demographic expansions by DNASP v5.0. Multiple methods were performed to understand the population genetic structure of *S. yangi*. Pairwise Fst [58] was examined using ARLEQUIN v3.5 [55] for each pair of the sampled populations; then, Mantel tests of the genetic distance [Fst/(1-Fst)] vs the geographical distance (ln km) based on mitochondrial genes (*COI + COII + Cytb + A6A8*) calculated with ZT v1.1 [59] were used to estimate the degree of isolation by distance.

### Divergence time estimation

The divergence times for the haplotype lineages were estimated using BEAST v1.8.1 [60] based on the combined mitochondrial genes data. The data were divided into two partitions, one composed of the protein*-*coding genes with substitution model HKY + I + G and the other containing the ribosomal RNA gene with HKY + G. Coalescent tree priors were set to the constant size model. Because no fossils are known for this lineage, it was not possible to calibrate the molecular clock using fossil-based minimum ages [29, 61, 62]. Thus, approximate divergence times were estimated using two previously proposed conventional mutation rates for the insect mitochondrial *COI* gene 2.3% (i.e., 0.0115 substitutions/site per lineage) and 3.54% (i.e., 0.0177 substitutions/site per lineage) per million years [63, 64]. Two runs were executed for 200 million generations, sampling every 20,000 generations and discarding the initial 25% as burn-in. Both the posterior distribution and the effective sample sizes (ESSs) from the MCMC output were measured by TRACER v1.5 [50]. TREEANNOTATOR v1.8.0 [60] was applied to obtaining a maximum credibility tree with the 95% highest posterior density (HPD) intervals and the annotation of mean node ages. After the analyses, all parameters were assessed to determine whether a sample size > 200 was obtained. The tree diagram with divergence time estimates was generated using FIGTREE v1.3.1 [65].

### Calling song structure comparison among populations

We obtained 23 acoustic samples of the male calling song in the field on 06 June, 2017 at the Chunshugou Valley in the Helan (HL) Mountains, Ningxia Hui Nationality Autonomous Region; 21 on 01 June, 2014 at Hancheng (HC), Shaanxi Province; 19 on 05 June, 2014 at Tongchuan (TC), Shaanxi Province; and 15 on 19 June, 2016 at Pingliang (PL), Gansu Province, China, at an average temperature ~ 30 °C. We used a linear PCM recorder (PCM-D100, Sony, China, frequency range of 20–20,000 Hz) for all acoustic recordings using two stereo microphones. The original stereo format (WAV) was converted to mono at a sampling rate of 44.1 kHz with 16 bits resolution. The analysis of acoustic properties was conducted using RAVEN PRO v1.4 (The Cornell Lab of Ornithology, Ithaca, NY, USA) and SEEWAVE v2.0.4 [66] which is a custom-made library of the R software platform [67]. Terminology adopted to describe the acoustic signals follows Puissant & Sueur [68].

Previous study has indicated that *S. yangi* males produce both timbal and stridulatory sounds for intraspecific communication [36]. Therefore, the frequency domain and duration of both timbal and stridulatory sounds of the four sampled populations were compared. Fast Fourier Transform (FFT) was applied to measuring dominant frequencies with 44.1 Hz precision. The one-way analysis of variance (ANOVA) was used to examine the dominant frequency and duration of different parts of the calling song among different populations, followed by the Student-Newman-Keuls test. Statistical analysis was undertaken with IBM SPSS Statistics v20.0 (IBM Corp, Armonk, NY). All statistical tests were two tailed, and *P* < 0.05 was considered significant.

## Results

### Sequence characterization

In total, 790 sequences of the six genes were obtained. The final alignment included 561 bp of *COI*, 699 bp of *COII*, 561 bp of *Cytb*, 657 bp of *A6A8*, 727 bp of *ITS1*, and 711 bp of *EF-1α.* The Chi-square test revealed no significant base composition heterogeneity among the populations for any gene fragment (*P* > 0.05). *COII* is the most variable gene, and most of the substitutions occurred in the third codon positions of this gene. The *ITS1* gene is more conserved than the mitochondrial genes. Within *S. yangi*, 5.46% of the sites in *COII* were parsimony-informative, compared to 0.18% in *EF-1α*.

### Phylogenetic relationships and population structure

**Fig. 2:**
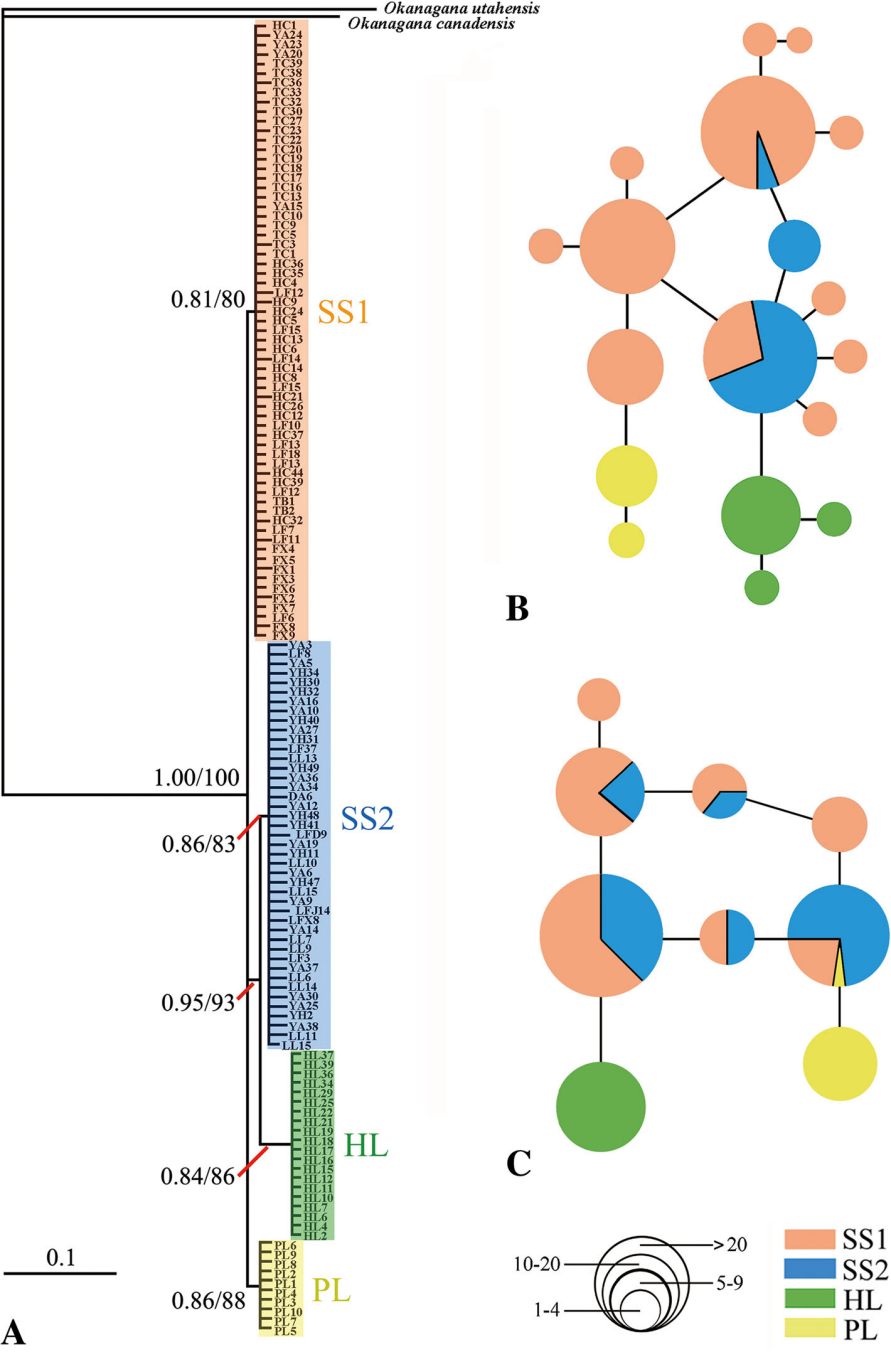
Phylogenetic tree and network profile. **a** phylogram reconstructed based combined mitochondrial (*COI* + *COII + Cytb + A6A8*) genes. Bayesian posterior probabilities/ ML bootstrap values are indicated near branches. **b** network of mitochondrial gene haplotypes of *S. yangi*. **c** network of nuclear gene haplotypes of *S. yangi*. The sizes of circles are proportional to haplotype frequency. Colours denote lineage membership and are the same as in **a**

**Fig. 3:**
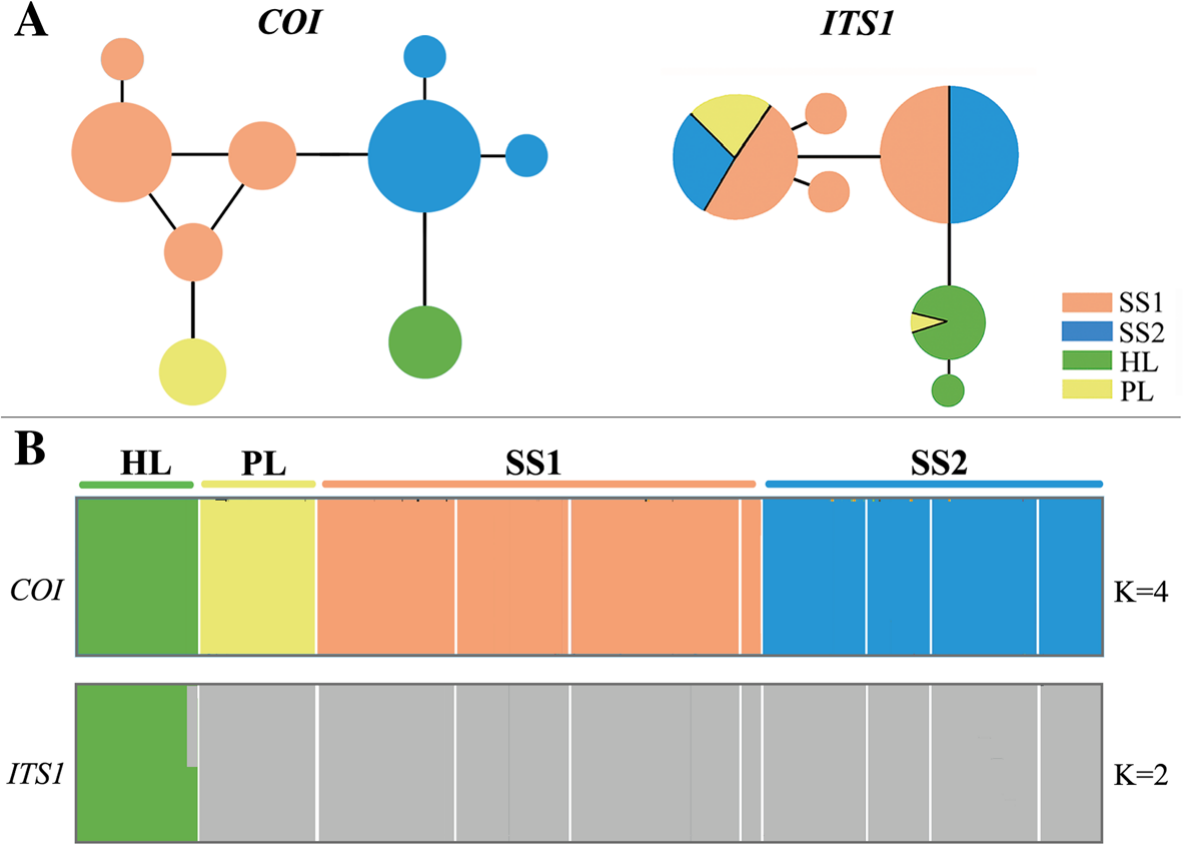
Population structure of *S. yangi* using the Network and Bayesian analysis of population structure (BAPS). **a** network of *S. yangi* based on *COI* and *ITS1* gene haplotypes. **b** Bayesian analysis of population genetic structure (BAPS) based on *COI* (K = 4) and *ITS1* (K = 2) sequences. Structure plots depicting population structure among samples from ten sites. Colours indicate different genetic clusters

ML and Bayesian phylogenies obtained from analysis of combined mtDNA (Fig. 2a) and nuDNA (Additional file 2: Figure S1) datasets were congruent (Additional file 3: Figure S2). Twenty-two haplotypes based on combined genes (mtDNA and nuDNA) were identified (Additional file 1: Table S2). HKY + G, HKY + G + I and GTR + I were the best-fit models for the partitions (*COI* + *COII* + *Cytb* + *A6A8*), (*EF-1α + ITS1*) and (*COI* + *COII* + *Cytb* + *A6A8* + *EF-1α + ITS1*), respectively. Both the phylogenetic trees and the network reveal low levels of genetic differentiation and a relatively simple phylogeographic structure for this species (Fig. 2). However, the analyses recovered two independent clades corresponding to the HL population and the PL population base on the combined mtDNA (Fig. 2a). Haplotype networks for mtDNA (Fig. 2b) and nuDNA (Fig. 2c) both show that groups SS1 and SS2 formed intermediate lineages between the more divergent and independent HL and PL lineages. The separate HL and PL clades were supported by Bayesian PP of 0.84 and 0.86, respectively, and ML bootstrap of 86 and 88%, respectively (Fig. 2a). In contrast, no distinct differentiation was found among the remaining populations distributed in the Loess Plateau and adjacent areas (i.e., in Shaanxi and Shanxi provinces), which formed two interlaced groups, viz SS1 and SS2 (Figs. 2a and 3a). Populations distributed in Shaanxi and Shanxi showed genetic affinity, but no clear association between the clades and geographic distribution was found. SS1 and SS2 included aggregations of individuals distributed in different localities and showed no clear biogeographic structural patterns.

AMOVA of *S. yangi* populations indicates that mitochondrial genetic variation among the 4 groups (HL, PL, SS1, SS2) was 89.04% (Fct = 0.00974, *P* < 0.0015), but only 6.42% (Fsc = 0. 00774, *P* < 0.0015) among populations within groups, and 4.54% (Fst = 0. 00044, *P* < 0.0015) within populations (Table 2). In contrast, AMOVA of the nuclear gene *ITS1* indicates that 79.33% (Fct = 0.20755, *P* < 0.0015) of genetic variation occurs within populations, but only 8.11% (Fst = 0.08049, *P* < 0.0015) among groups (Table 2). Based on the combined nuclear genes (*EF-1α + ITS1*), AMOVA indicates 85.46% (Fct = 0.17504, *P* < 0.0015) of genetic variation occurs within populations (Table 2).

**Table 2:** Analysis of molecular variance (AMOVA) results based on the mitochondrial genes and nuclear genes

Genes	Source of variation	Fixation indices	*P*-value	Variance components	Percentage of variation
*COI + COII+* *Cytb + A6A8*	Among groups	Fct = 0.00974	0.00035	7.02571 Va	89.04%
	Among populations within groups	Fsc = 0. 00774	0.00058	1.05875 Vb	6.42%
	Within populations	Fst = 0. 0044	0.00000	2.14570 Vc	4.54%
	Total		0.00093	10.23016	
*COI*	Among groups	Fct = 0.84075	0.00000	18.02355 Va	84.17%
	Among populations within groups	Fsc = 0.33091	0.00490	1.12977 Vb	5.17%
	Within populations	Fst = 0.89343	0.00000	2.28445Vc	10.66%
	Total		0.00490	21.43777	
*ITS1*	Among groups	Fct = 0.08049	0.00000	0.06788 Va	8.11%
	Among populations within groups	Fsc = 0.13758	0.00000	0.10618 Vb	12.56%
	Within populations	Fst = 0.20755	0.00000	0.66475 Vb	79.33%
	Total		0.00000	0.83881	
*EF-1α + ITS1*	Among groups	Fct = 0.06207	0.00000	0.18647 Va	4.21%
	Among populations within groups	Fsc = 0.11452	0.00000	0.24571Vb	10.33%
	Within populations	Fst = 0.17504	0.00000	0.30141 Vb	85.46%
	Total		0.00000	0.73369	

The median-joining network of *COI* haplotypes also revealed four groups, viz., HL, PL, SS1 and SS2 (Fig. 3a). AMOVA of *S. yangi* populations indicates that mitochondrial genetic variation among the 4 groups is 84.17% (Fct = 0.84075, *P* < 0.0015). Similarly, the BAPS clustering analysis for the *COI* gene supported four main groups within the ten populations (K = 4; group 1: HL; group 2: PL; group 3: SS1 (FX, HC, LF, TB, TC, YA); group 4: SS2 (HY, LF, LL, YA); Additional file 1: Table S3; Fig. 3b). BAPS analysis for the gene *ITS1* revealed two groups (K = 2; Additional file 1: Table S3; Fig. 3b). A visual assessment of the BAPS results shows that the HL population forms a single distinct cluster, while the remaining populations form another with apparent admixture (Fig. 3b).

### Demographic analysis

Mitochondrial genetic diversity indices for each population are presented in Table 3. Haplotype diversity is relatively low, ranging from 0.968 (LF) to 0.216 (TC), and nucleotide diversity ranges from 0.00191 (LF) to 0.00009 (TC). Neutrality tests conducted for the 10 populations of *S. yangi* for the combined mtDNA dataset (Table 3) indicate that the HL population shows significant negative values for both Fu’s F (*p* < 0.05) and Tajima’s D (*p* < 0.05) statistics. These results significantly reject the hypothesis of neutral evolution in this population, indicating that recent population expansion has occurred in the Helan Mountains and/or adjacent areas. The unimodal curve of the mismatch distribution supports the same inference of a population expansion (Additional file 4: Figure S3).

**Table 3:** Genetic diversity and neutrality tests calculated for populations

Population	NS	Nh	*H* ± SD	π ± SD	Tajima’ D	Fu’s Fs
FX	18	2	0.278 ± 0.037	0.00087 ± 0.00033	0.77973	−1.691
HC	20	3	0.424 ± 0.061	0.00142 ± 0.00016	0.26627	0.158
HL	20	3	0.298 ± 0.018	0.00017 ± 0.00003	−1.8622*	−2.005*
LF	20	9	0.968 ± 0.026	0.00191 ± 0.00023	−0.96593	−10.779
LL	20	3	0.605 ± 0.102	0.00091 ± 0.00025	−1.20798	−1.352
PL	17	2	0.233 ± 0.180	0.00044 ± 0.00017	0.02107	−0.742
TB	2	1	–	–	–	–
TC	18	3	0.216 ± 0.124	0.00009 ± 0.00006	−1.50776	−1.744
YA	20	3	0.576 ± 0.124	0.00091 ± 0.00028	−0.10574	−0.022
YH	10	2	0.367 ± 0.086	0.00139 ± 0.00037	−0.94371	−1.744

*NS* number of samples, *Nh* number of haplotypes, *H* haplotype diversity, π nucleotide diversity, SD standard deviation, *Significant value

The mean Fst ranged from 0.0018 (LL population) to 0.0735 (HL population) (Table 4). Significantly, genetic divergence was found between the HL population and all other populations with the maximum mean differentiation value (mean Fst ¼ 0.0646, 0.0601–0.0735). Similarly, significant genetic differentiation was also found between the PL population and other populations (mean Fst ¼ 0.0541, 0.0408–0.0601). The Mantel test yielded an r value of 0.0289 for combined mitochondrial gene data (*P* = 0.0012) (Additional file 5: Figure S4), indicating a significant correlation between genetic and geographic distances in *S. yangi* populations.

**Table 4:** Genetic differentiation among populations of *S. yangi* based on mitochondrial (*COI* + *COII* + *Cytb* + *A6A8*) genes

Population	FX	TB	TC	HC	YA	LL	LF	LFD	PL	HL
FX										
TB	0.0101									
TC	0.0101	0.0091								
HC	0.0121	0.0111	0.0124							
YA	0.0166	0.0116	0.0090	0.0037						
LL	0.0141	0.0121	0.0084	0.0068	0.0018					
LF	0.0181	0.0141	0.0099	0.0035	0.0235	0.0106				
YH	0.0155	0.0135	0.0096	0.0068	0.0158	0.0086	0.0019			
PL	0.0578^*^	0.0408	0.0569^*^	0.0598^*^	0.0599^*^	0.0488	0.0485	0.0573^*^		
HL	0.0676^*^	0.0656^*^	0.0607^*^	0.0735^*^	0.0670^*^	0.0638^*^	0.0613^*^	0.0617^*^	0.0601^*^	

^*^Indicates significant difference at *p* < 0.05 level

### Genetic distances

Pairwise corrected genetic distances for mitochondria DNA of *S. yangi* and outgroup species are shown in Additional file 1: Table S4. Intraspecific genetic distances of *S. yangi* (0.001–0.018) are distinctly lower than distances between *S. yangi* and the outgroup species (0.108–0.148), without overlap. Except for comparisons between the HL population and other populations, most pairwise comparisons show very low genetic distance values, suggesting these populations have a low level of genetic diversity (Additional file 1: Table S4). The highest intraspecific divergence values (0.006–0.018) are those between the HL population and the remaining populations. The intraspecific divergence values between the PL population (the other independent lineage) and the remaining populations, except for the HL population, increase along with the increase in geographic distance, varying from 0.005 to 0.009.

### Estimation of divergence time

**Fig. 4:**
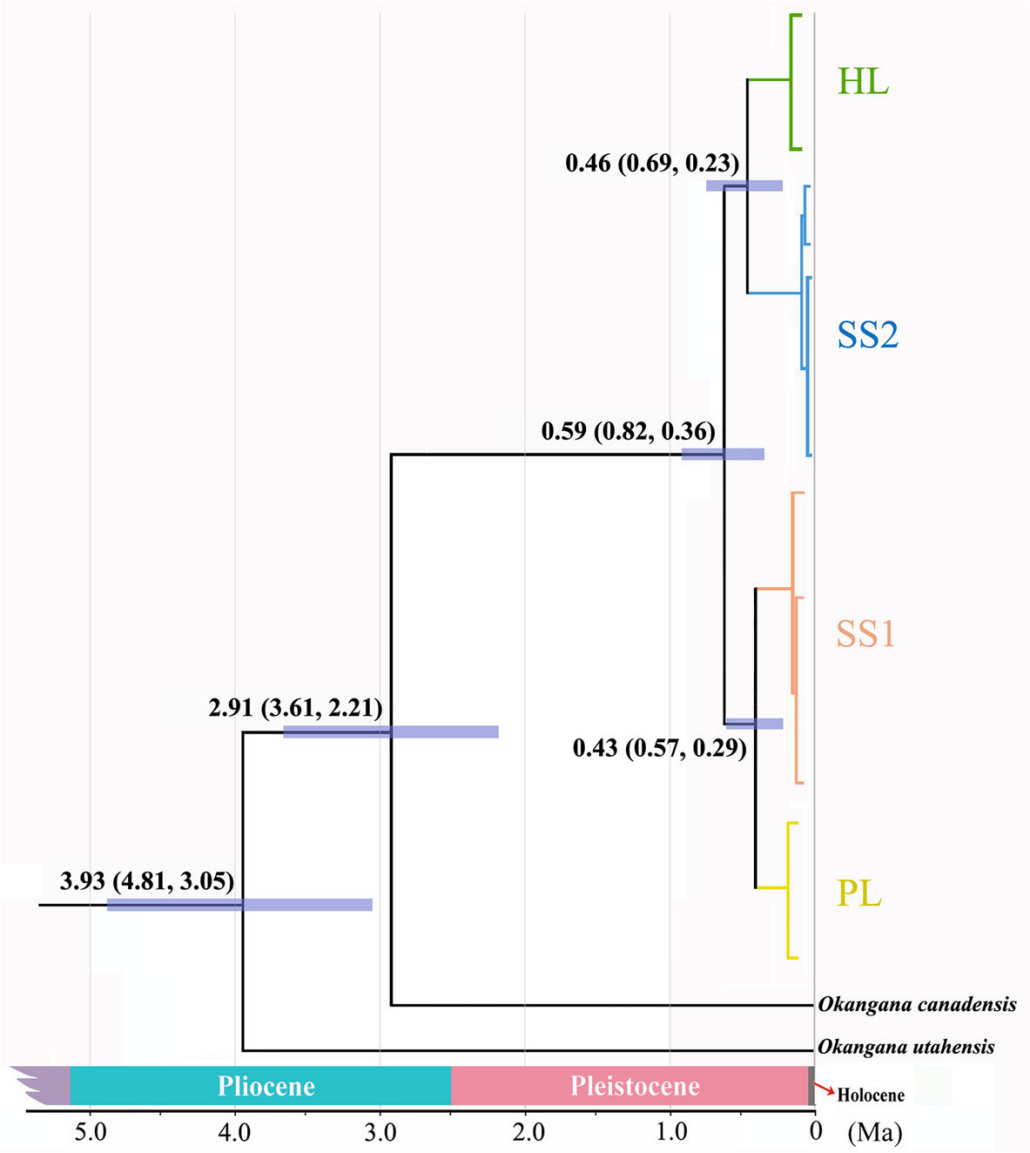
Chronogram of *S. yangi* based on mtDNA data, using the conventional divergence rate of 3.54% per million years. Estimates of divergence time are shown at nodes above branches. Divergence times at or below approximately 0.01 Ma are not shown

The divergence-time chronogram places the split between *Okanagana utahensis* and the remaining species at a mean age of ~ 3.93 Ma (95% highest posterior density interval, HPDI, 3.05–4.81 Ma), when the conventional insect mtDNA divergence rate of 3.54% per million years is used (Fig. 4). The estimated divergence time between *S. yangi* and *Okanagana canadensis* was ~ 2.91 Ma (95% HPDI, 2.21–3.61 Ma) (Fig. 4). Estimated times of divergence were ~ 0.59 Ma (95% HPDI, 0.82–0.36 Ma) between populations (HL + SS2) and (PL + SS1). HL and SS2 diverged at ~ 0.46 Ma (95% HPDI, 0.23–0.69 Ma), and a divergence time of 0.43 Ma (95% HPDI, 0.29–0.43 Ma) was estimated for the split of PL and SS1 (Fig. 4). When the alternative conventional mtDNA divergence rate of 2.3% per million years was used to construct the chronogram (Additional file 6: Figure S5), the estimated times of divergence were ~ 0.29 Ma (95% HPDI, 0.19–0.39 Ma) between populations (HL + SS2) and (PL + SS1); HL and SS2 diverged at ~ 0.20 Ma (95% HPDI, 0.13–0.27 Ma); and a divergence time of 0.19 Ma (95% HPDI, 0.12–0.26 Ma) was estimated for the split of PL and SS1 (Additional file 6: Figure S5).

### Calling song structure comparison among populations

**Fig. 5:**
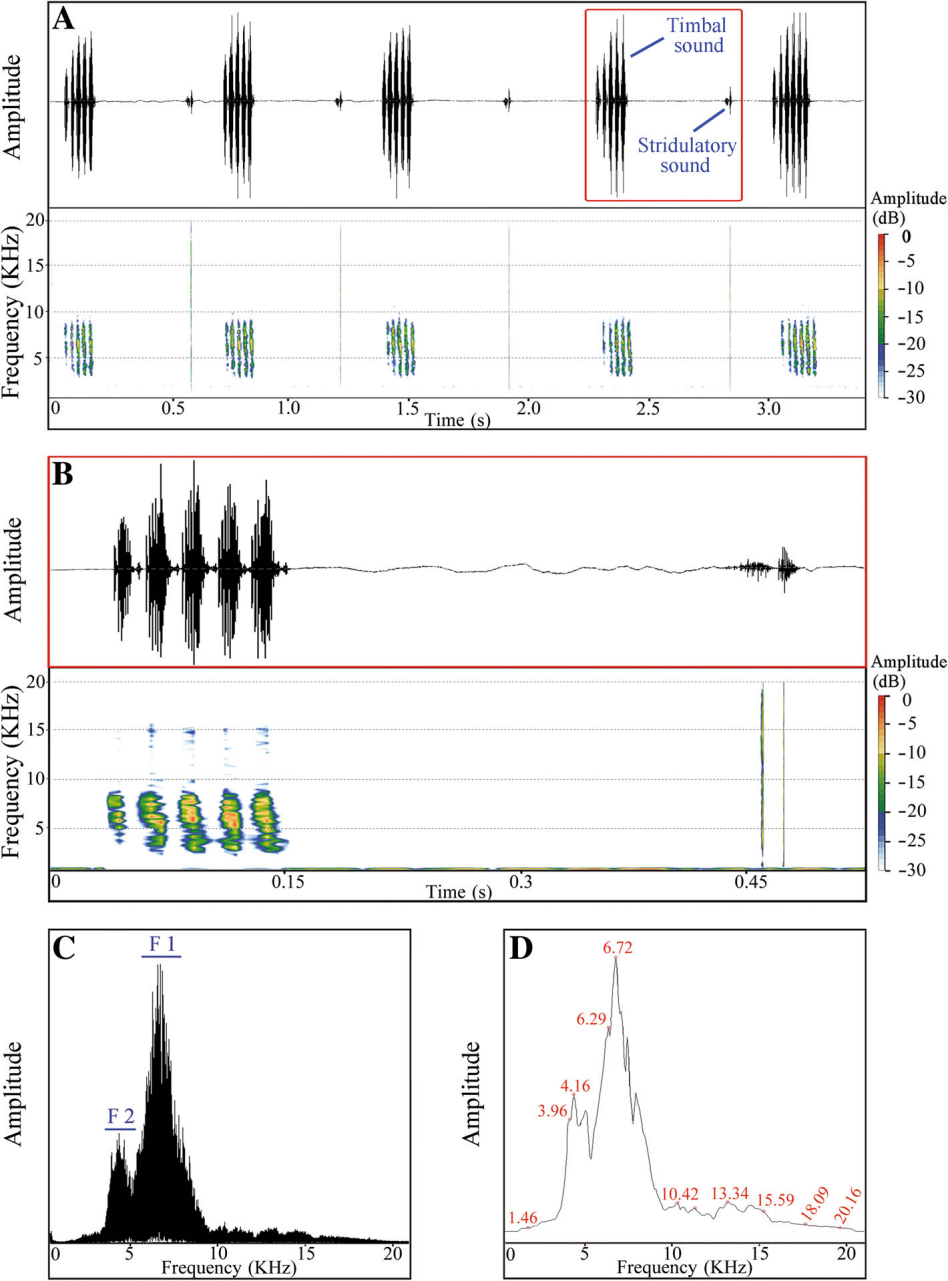
Acoustic analyses of the male calling song structure of *S. yangi* from Helan Mountains, Ningxia (HL). **a** oscillogram and spectrogram of the timbal and stridulatory sounds shown alternately (i.e., upward and downward echemes). **b** detailed oscillogram and spectrogram of timbal and stridulatory sounds (marked by the red box in **a**). **c**, **d** power frequency spectrum with overlay of 174 spectra computed in the middle of the signal showing dominant frequencies marked by F1 and F2

**Fig. 6:**
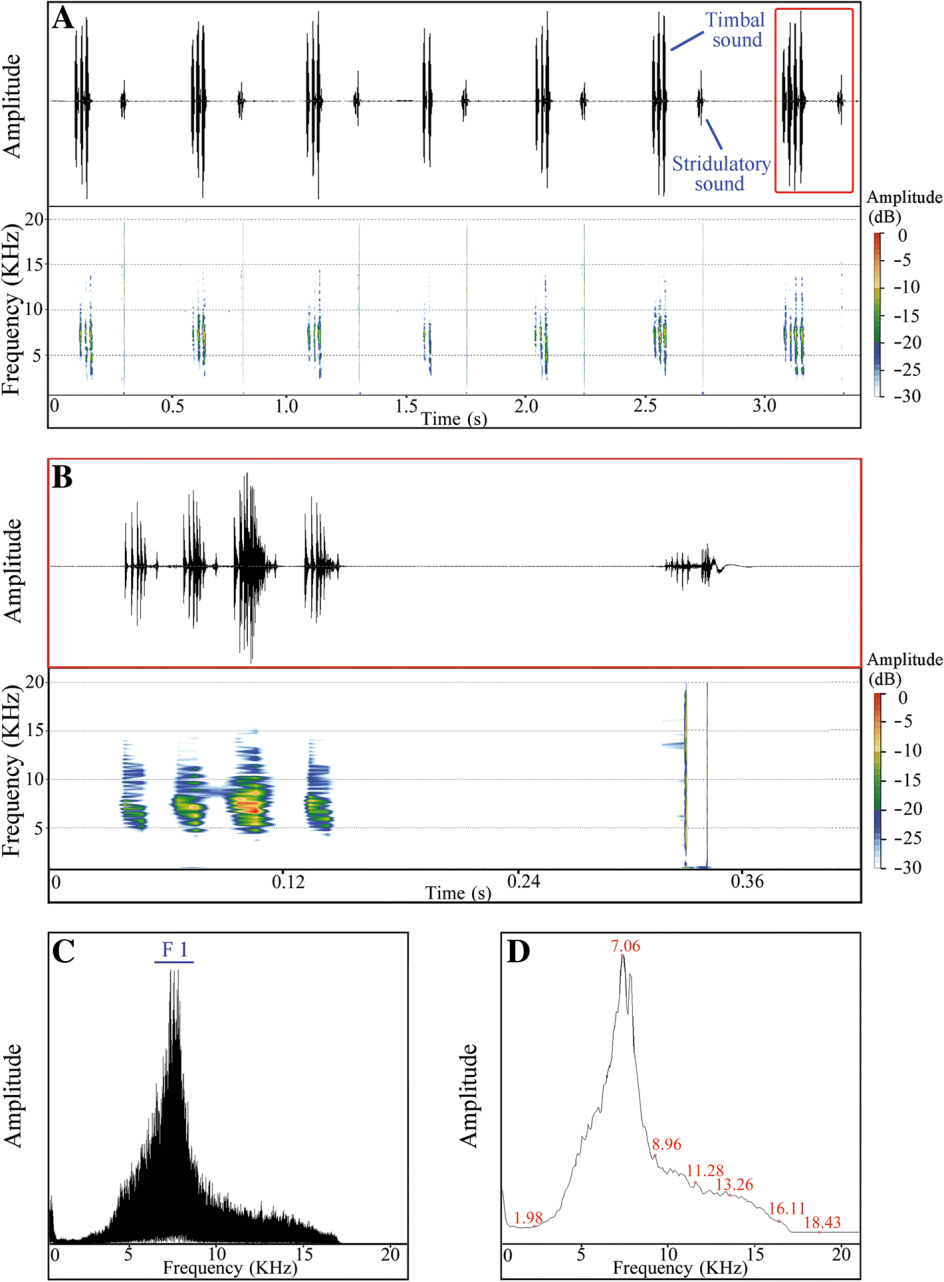
Acoustic analyses of the male calling song structure of *S. yangi* from Hancheng (HC). **a** oscillogram and spectrogram of the timbal and stridulatory sounds shown alternately (i.e., upward and downward echemes). **b** detailed oscillogram and spectrogram of timbal and stridulatory sounds (marked by the red box in **a**). **c**, **d** power frequency spectrum with overlay of 174 spectra computed in the middle of the signal showing dominant frequencies marked by F1

Calling song patterns indicate that male *S. yangi* produce timbal and stridulatory sounds alternately. In the HL population, the calling duration of an entire song consisting of one timbal sound and one stridulatory sound is approximately 451.1 ± 40.7 ms (mean ± SD; range = 410.4–491.7 ms) (*N* = 23) (Fig. 5a, b). Each timbal sound is composed of ~ 3–7 echemes (Fig. 5a, b). The power spectrum shows two main parts (F1 and F2) in the frequency domain of each timbal sound (Fig. 5c, d): F1, possessing the maximum amplitude, is between 6.29~7.15 KHz with a peak around 6.72 KHz; F2 is between 3.96~4.36 KHz with a peak around 4.16 KHz. The spectral characteristics of the remaining populations are very similar (Fig. 6, Additional files 7 and 8: Figures S6 and S7), but significantly different from that of the HL population. Specifically, the duration of an integrated calling song comprising one timbal sound and stridulatory sound is approximately 324.1 ± 36.1 ms (mean ± SD; range = 288.0–360.2 ms) (*N* = 21), and each timbal sound is made up of ~ 2–4 echemes (Fig. 6a, b). In contrast, the power spectrum in the remaining populations shows only one main part (F1) in the frequency domain of each timbal sound (Fig. 6c, d), which is between 6.61~8.09 KHz with a peak around 7.35 KHz.

**Fig. 7:**
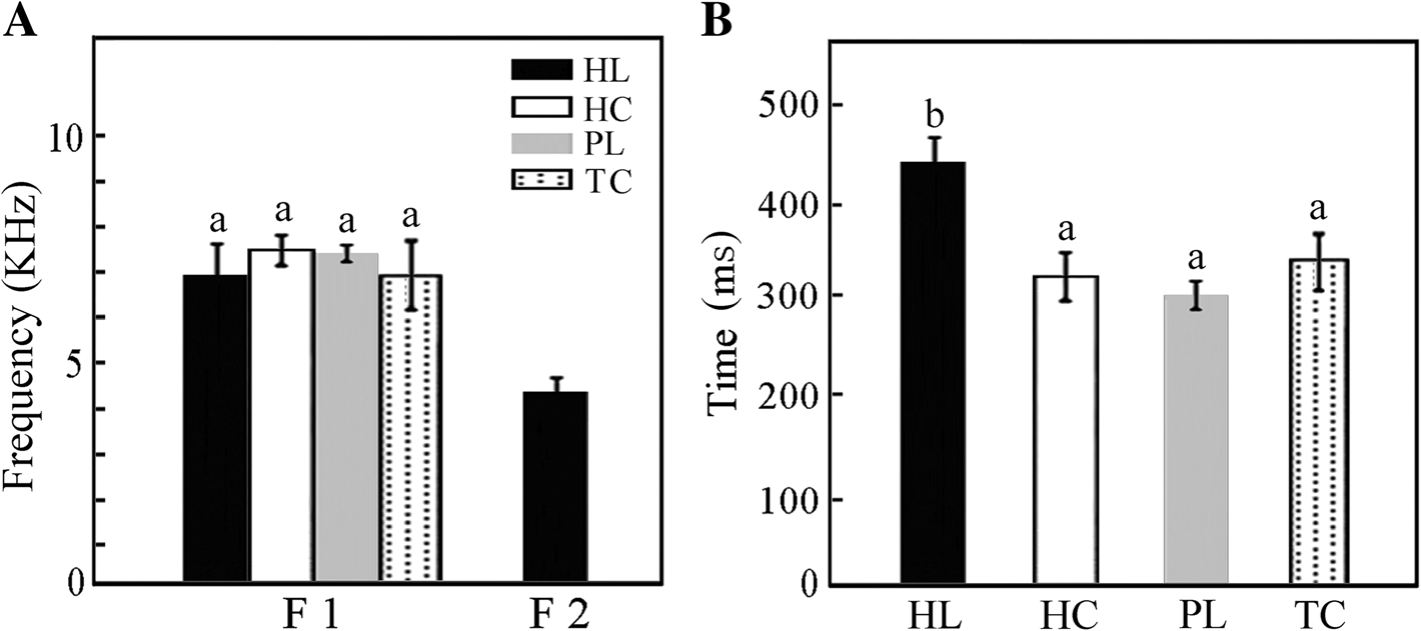
Comparison of calling song structure of populations of *S. yangi*. **a** comparison of power frequency spectrum of F1 and F2 of four populations (mean ± SD). **b** comparison of duration from a timbal sound to a stridulatory sound of four populations (data show mean ± SD)

The frequency domain of F1 of each timbal sound among the four representative populations (i.e., HL, HC, TC and PL) shows no significant difference (*P* > 0.05) (Fig. 7a), which is obviously higher than the exclusive dominant frequency F2 found in the HL population (Fig. 7a). The durations of both the timbal and stridulatory sounds of the HL population are remarkably different (*P* < 0.05) from those of the remaining populations (Fig. 7b). The HL population differs in calling song structure with respect to the dominant frequency of the timbal sound and the duration of both the timbal and stridulatory sounds (*P* < 0.05).

## Discussion

*Subpsaltria yangi* is the only species of the subfamily Tettigadinae known from China [32]. This rare species was originally reported from the foothills of the Qinling Mountains [33]. It was thought to be extinct after intensive field investigations in recent decades [36]. After the discovery of a population of *S. yangi* from the Helan Mountains in June 2011, a few more populations were discovered using our special acoustic playback method [35]. Our distribution data for *S. yangi* suggest that this species is restricted to the Loess Plateau and adjacent areas of northwestern China. Previous studies show that *S. yangi* is similar to cicadas of the genus *Paharia* Distant in morphology and habitat (i.e., deserts and semi-deserts) [69–71]. *Paharia* cicadas are patchily distributed primarily in the Middle East [72]. Similarly, *S. yangi* occurs locally in patches of suitable habitat. The distribution patterns of *Subpsaltria*, *Paharia* and their relatives suggest that the evolution of this tribe has been closely tied to geographic range contraction resulting from historical climatic change [72].

Our study based on combined maternally inherited mitochondrial DNA and bi-parentally inherited nuclear genes provided a comprehensive framework to analyse the phylogeography and speciation of *S. yangi*. The population structure of *S. yangi* was revealed to comprise one large unit with low genetic diversity hierarchy but obvious differentiation was revealed between the HL population and other populations based on analyses of genetic distance, haplotype networks, and the calling song structure of males. The deserts and semi-deserts surrounding the Helan Mountains were previously shown to represent a major dispersal and climatic barrier [24]. Such barriers appear to have promoted divergence of the HL lineage from other lineages of *S. yangi*. *Subsaltria yangi* may, therefore, represent a new example of the early stage of speciation in insects. Cases such as this, where speciation occurs through geographic isolation and genetic differentiation of a peripheral population have been referred to as “budding speciation” [73].

Quaternary glaciations are often regarded as a major factor in forming the biodiversity of various extant species [12]. Our divergence-time estimates indicate that the geographical distribution and genealogical divergences of *S. yangi* are consistent with the scenario of glacial refugia and interglacial climate oscillation in northwestern China. Our estimates (Fig. 4) suggest that divergence between the HL population and other populations occurred in the Pleistocene (0.15–2.5 Ma). In addition, our analysis shows that some divergence occured among populations in different habitats in spite of considerable gene flow [74], possibly due at least in part to habitat and host plant specialization. The HL population occurs at elevations of 1400–1600 m above the sea level, while other populations occur at elevations below 1000 m (Fig. 1). The Helan Mountains are located in the transition zone between the temperate and desert steppe where fauna and flora are complex in this monsoon boundary zone [20, 75]. Furthermore, the HL population feeds on *Ephedra lepidosperma* (Ephedraceae), an endemic plant in the Helan Mountains, but other populations except the PL population occur in the Loess Plateau and adjacent areas and feed on *Zizyphus jujuba* Mill. var. *spinosa* (Bunge) Hu ex H. F. Chow. Hence, we hypothesise that ecological differences among habitats, coupled with the geographical isolation, might have reinforced genetic and behavioral divergence of the HL population.

Previous phylgeographic studies indicated that geographic barriers, including mountains, water bodies, deserts or inhospitable terrain, can drive speciation in geographically isolated populations [76, 77]. For example, genetic divergence in populations of many other insects, e.g., the beetle *Carabus solieri* [78], the butterfly *Parnassius mnemosyne* [79], the moth *Apocheima cinerarius* [8] and the ant *Acromyrmex striatus* [80], were revealed to be triggered by geographical barriers, and the contribution of geographic isolation is apparent in recent speciation events. Our study also found that geographical barriers (viz., the deserts and semi-deserts surrounding the Helan Mountains) played an important role in shaping the phylogeographical structure of *S. yangi*. Our phylogeny, genetic distances, BAPS and haplotype networks of *S. yangi* all indicate that a major genetic gap exists between the PL population and remaining individuals (Figs. 2 and 3; Table 4; Additional file 1: Table S4). Most likely, this was caused by the disruption of gene flow by the Liupan Mountains. During the late-Cenozoic, approximately 3.6 Ma, the Liupan Mountains rose to elevations of about 2600 m [81] which, together with the Qinling Mountains, obstructed the westward flow of the wet southeastern monsoon, and increased the aridity of western China [81, 82]. The climate of western China was dry and cold during the early late Pleistocene [83]. Accordingly, we presume that the independently evolved PL lineage is also associated with the natural barrier formed by the Liupan Mountains. During our field investigations, the PL population appears mostly to utilize *Prunus mongolica* Maxim as a host plant. This may represent another example of a host shift in *S. yangi*, similar to that already described for the HL population, and merits further study.

Ecological divergence and geography can both play important roles in speciation [84]. Previous studies have indicated that ecological factors may play an even more important role than geographical factors in shaping population structure [85]. Ecological specialization yielded suites of adaptive morphological traits and led to population divergence in animals like *Timema* walking-stick insects [86], *Heliconius* butterflies [87], *Loxia* crossbills [88], and *Pundamilia* cichlids [89]. In the case of *S. yangi*, although the deserts and semi-deserts surrounding the Helan Mountains represent a major dispersal and climatic barrier between the HL population and the remaining populations of *S. yangi*, we infer that the unique host, habitat and ecological environments of the HL population also contributed to divergence of this lineage yielding phenotypical differences from other populations. Our results also indicate that the PL population and the SS1 and SS2 groups represent different genetic lineages, but these populations do not show significant difference in the calling song structure (Figs. 4 and 7). This may also be consistent with a more recent divergence of the PL population compared to the HL population, which possibly indicates an even earlier stage of speciation than the HL population. The difference in calling song between HL and the other populations may have been a result from previous contact between HL and the other populations due to temporary merging of suitable habitats during a previous glacial cycle. Other studies (e.g., of frogs) have shown that when previously allopatric populations come into contact, they sometimes acquire additional pre-copulatory barriers to gene flow, such as different courtship songs [3]. Such behavioral divergence often does not occur as long as populations remain allopatric. This hypothesis for *S. yangi* needs to be tested by further analyses based on integrating phylogenetic and network approaches. Although the gene flow between the PL population and the SS1 as well as the SS2 groups may have been disrupted by the Liupan Mountains, the habitats occupied by these isolated populations are similar to each other. Previous studies demonstrated that, although time spent in isolation should be the primary factor in predicting phenotypic differentiation of ecologically similar allopatric populations [90], phenotypic differentiation in similar environments may take a long time [91]. The rate of phenotypic divergence among the PL population and the SS1 and SS2 populations may be lower due to lower selection pressure compared to that which occurred in the HL population.

Rivers have acted as substantial barriers to gene flow within some Chinese insects [92], plants [93, 94], and even birds [95, 96]. Thus, we expected to find genetic divergence between populations of *S. yangi* occurring in Shaanxi and Shanxi provinces because they are located on the opposite sides of the Yellow River. However, samples distributed in Shaanxi and Shanxi showed no obviously genetic differentiation (Fig. 2). This is concordant with prior studies [24, 97] that showed the Yellow River did not act as a natural barrier to gene flow in some insects.

The cicada *S. yangi* has an unusual phonation mode among cicadas in that, besides the timbal organs of males, well-developed stridulatory organs are found in both sexes [35]. Cicadas are well known to have species-specific calling songs that enable individuals of sympatric species to distinguish among potential mates, and the calling song of male cicadas is routinely used as a taxonomic character [98]. Moreover, divergence in acoustic signals has a significant effect on speciation [17]. The underlying mechanisms driving divergence of acoustic traits and their consequences in *S. yangi* speciation remain poorly understood. Our study revealed that the calling song structure of the HL population distinctly differs in frequency domain and duration from that of the remaining populations (Fig. 7). The acoustical difference of the male calling song between the HL population and other populations, coupled with the results of molecular phylogenetic analyses (Figs. 2 and 3; Additional files 2 and 3: Figures S1 and S2), suggests that the isolated HL population has evolved into a distinct independent lineage. This suggests that premating isolation may evolve rapidly and result in rapid speciation in species with low levels of general genetic differentiation.

Finally, human activities are important factors that have shaped the current genetic structure of many species [99, 100]. Tainaka et al. [101] illustrated that frequent speciation or extinction may occur in communities undergoing rapid environmental change. Habitat fragmentation and destruction of host-plants by human activities, particularly agricultural practices, is a critical threat to populations of *S. yangi* [36]. Therefore, assessment of habitat conditions and conservation needs of this rare species should be conducted.

Estimated divergence time between *S. yangi* populations differed sharply depending on which conventional mtDNA divergence rate was used, i.e., 2.3% versus 3.54% per million years (Fig. 4, Additional file 6: Figure S5). When the 3.54% divergence rate was applied, the estimated age was congruent with the geological events that separated the main areas occupied by different populations of *S. yangi* and with the results of phylogeographic analysis based on combined mitochondrial gene data (Fig. 4). Nevertheless, the 2.3% standard clock appears to be satisfactory to estimate ages in many species based on only cytochrome oxidase I (*COI*) sequence data [63]. Preference for either of the two alternative conventional substitution rates (2.3 and 3.54%) has tended to differ depending on the particular geologic events used in calibrating chronograms [64]. Further studies should be done to test the accuracy of divergence times in cicadas via applying alternative substitution rates and calibration methods.

Hierarchical analysis of molecular variance (AMOVA) [54] analyses population subdivision using F-statistics by measuring correlation between genetic variation which can be impacted by several evolutionary factors such as mutation rates or migration [102]. Unfortunately AMOVA requires an a priori definition of the hierarchical structuring of populations, which might introduce bias [54]. The AMOVA results may also differ between the nuclear and mitochondrial DNA [54, 103], as occurred in our study. The hypothesis of significant genetic structure among *S. yangi* haplogroups is well supported (Table 2), and 89.04 and 4.21% of the mtDNA and nuDNA genetic variation among groups were revealed, respectively. The analyses indicated that partitioning into the major haplogroups explains 89.04% of the overall mtDNA variability and corresponds to a highly significant fixation index (*p* < 0.0015; Table 2). The AMOVA results for the *COI* gene show that mitochondrial genetic diversity is mostly explained by differences among groups, while for the nuclear gene *ITS1*, more genetic variation is found within populations (Table 2). The divergence between the four lineages of *S. yangi* indicates restricted gene flow and long-term isolation (Figs. 2 and 3), which should be caused by geographical barriers and the low dispersal ability of the species [104]. In contrast, AMOVA of the nuDNA data showed moderate, statistically significant nuclear allele divergence among the ten populations, but did not show statistically significant regional differences when we divided the populations into two regions. Nevertheless, our BAPS and network analyses identified two nuDNA groups (Fig. 3). We speculate that lower evolutionary rates may be the main cause of relative homogeneity in the nuclear genes [105, 106]. The presence of unique haplotypes within each lineage indicates some degree of genetic differentiation of the nuclear genes has occurred between the two lineages and suggests that interbreeding among lineages is low, but further studies are needed to test this hypothesis.

Habitat fragmentation and desertification resulting from Quaternary glaciations and human activities are the most likely factors that yielded the observed genetic diversity and population differentiation in *S. yangi*. Ecological differences in habitats, coupled with the geographical isolation and diet shifts might also have contributed to the divergence of the HL and PL populations from other populations and may represent early stages of speciation. Further analyses on divergence time of this species based on more molecular loci are required to test this hypothesis. Our results improve the understanding of genetic differentiation, acoustic signal diversification and phylogeographic relationships of this rare species of conservation concern, and inform future studies on population divergence, speciation and phylogeography of other insects with a high degree of endemism in the Helan Mountains and adjacent areas.

## Additional files

Additional file 1: **Table S1.** Primer names, sequences used in PCR reactions of genes sequenced. **Table S2.** List of grouping and haplotypes about populations with mtDNA and nuDNA genes. **Table S3.** Population clusters of *S. hilpa* found by BAPS. **Table S4.** Intraspecific and interspecific genetic distance of *S. yangi* and other related species based on (*COI* + *COII + Cytb + A6A8*) gene. (DOCX 27 kb)

Additional file 2: **Figure S1.** Phylogram reconstructed based (*EF-1α + ITS1*) genes. Bayesian posterior probabilities and ML bootstrap values are indicated near tree branches. (TIF 1020 kb)

Additional file 3: **Figure S2.** Phylogram reconstructed based (*COI* + *COII + Cytb + A6A8 + EF-1α + ITS1*) genes. Bayesian posterior probabilities and ML bootstrap values are indicated near tree branches. (TIF 1513 kb)

Additional file 4: **Figure S3.** Pairwise mismatch distribution of HL population. X axis: Pairwise Differences. Y axis: Frequency. Obs means the observed distribution of pairwise difference. Exp means the expected equilibrium distributions. (TIF 1187 kb)

Additional file 5: **Figure S4.** Scatter plots of genetic distance vs. geographical distance for pairwise population comparisons (both analyses are calculated from 100,000 randomizations). (TIF 206 kb)

Additional file 6: **Figure S5.** The divergence time analysis of *S. yangi* based mtDNA gene, using the rate of 2.3% per million years. Estimates of divergence time are shown at nodes above branches. The divergence times occurred at approximately 0.01 Ma and under 0.01 Ma are not shown. (TIF 1010 kb)

Additional file 7: **Figure S6.** Acoustic analyses of the male calling song structure of *S. yangi* from Pingliang (PL). A, oscillogram and spectrogram of the timbal and stridulatory sounds were produced alternately (i.e., upward and downward echemes). B, detailed oscillogram and spectrogram of timbal and stridulatory sounds (marked by the red box in A). C, D, power frequency spectrum of the signal showing dominant frequencies marked by F1. (TIF 2804 kb)

Additional file 8: **Figure S7.** Acoustic analyses of the male calling song structure of *S. yangi* from Tongchuan (TC). A, oscillogram and spectrogram of the timbal and stridulatory sounds were produced alternately (i.e., upward and downward echemes). B, detailed oscillogram and spectrogram of timbal and stridulatory sounds (marked by the red box in A). C, D, power frequency spectrum of the signal showing dominant frequencies marked by F1. (TIF 3031 kb)

## Abbreviations

A6A8: ATPase subunits
AMOVA: A three-level hierarchical analysis of molecular variance
BAPS: Bayesian analysis of population structure
BI: Bayesian analysis
BIC: Bayesian Information Criterion
BS: Bootstrap support value
COI: Cytochrome c oxidase subunit I
COII: Cytochrome c oxidase subunit II
Cytb: Cytochrome b
DNA: Deoxyribonucleic acid
EF-1α: Elongation factor-1 alpha gene
ESSs: Effective sample sizes
FFT: Fast Fourier Transform
HPD: Highest posterior density
ITS1: Internal transcribed spacer 1
K2P: Kimura two-parameter
MCMC: Markov chain Monte Carlo
ML: Maximum likelihood
mtDNA: Mitochondrial DNA
nuDNA: Nuclear DNA
PCR: Polymerase chain reaction
PP: Bayesian posterior probabilities
PSRF: Potential Scale Reduction Factor
QTP: Qinghai-Tibet Plateau
TBR: Tree bisection-reconnection
