# Supplementary material for: Genetic divergence, population differentiation and phylogeography of the cicada Subpsaltria yangi based on molecular and acoustic data: an example of the early stage of speciation?
Source: BMC Evol Biol. 2019 Jan 8;19:5. doi: 10.1186/s12862-018-1317-8 (PMC6323834; doi:10.1186/s12862-018-1317-8)
Supplement: Supplementary file 1 — Table S1. Primer names, sequences used in PCR reactions of genes sequenced. Table S2. List of grouping and haplotypes about populations with mtDNA and nuDNA genes. Table S3. Population clusters of S. hilpa found by BAPS. Table S4. Intraspecific and interspecific genetic distance of S. yangi and other related species based on (COI + COII + Cytb + A6A8) gene. (DOCX 27 kb) [file 12862_2018_1317_MOESM1_ESM.docx]

**Additional file 1**

**Table S1. Primer names, sequences used in PCR reactions of genes sequenced**

| Primer for gene | Primer name | Primer sequence 5’–3’ | Reference |
| --- | --- | --- | --- |
| *COI* | LCO1490 | GGTCAACAAATCATAAAGATATTGG | Simon et al.  [1] |
|  | HCO2198 | TAAACTTCAGGGTGACCAAAAAATCA |  |
| *COII* | COII-3037 | TAGTATGGCAGATTAGTGCAATGAA | Zahniser et al. |
|  | COII | CCRCAAATTTCWGARCATTGACCA | [2] |
| *Cytb* | CB1 | TATGTACTACCATGAGGACAAATATC | Simon et al.  [1] |
|  | CB2 | ATTACACCTCCTAATTTATTAGGAAY |  |
| *A6A8* | A6A8-F | TGCTGCGGGTACTGGTGAAT | Marshall et al.  [3] |
|  | A6A8-R | ACACCAGTTTCAACTCTGCC |  |
| *EF-1α* | EF1-PA-f650 | TGCTGCGGGTACTGGTGAAT | Arensburger et al. [4] |
|  | EF1-N-1419 | ACACCAGTTTCAACTCTGCC |  |
| *ITS1* | CAS5p8sB1d | ATGTGCGTTCRAAATGTCGATGTTCA | Ji et al. [5] |
|  | CAS18sF1 | TACACACCGCCCGTCGCTACTA |  |

**Table S2. List of grouping and haplotypes about populations with mtDNA and nuDNA genes**

| Population | *COI+COII+Cytb+A6A8* | *COI+COII+Cytb+*  *A6A8+EF-1α+ITS1* | *ITS1* | *EF-1α+ITS1* |
| --- | --- | --- | --- | --- |
| FX | H2, H4 | H1, H2, H3 | H3 | H1, H6 |
| HC | H1, H2, H3 | H4, H7, H8, H9, H11, H12 | H1 | H1, H2, H5, H8 |
| HL | H6, H7, H8 | H21, H22 | H2, H7 | H3 |
| LF | H1, H2, H3, H4, H9, H13, H14, H16, H17 | H7, H8, H9, H10, H15, H16, H17, H18, H19, H13 | H1, H3 | H4, H5, H6 |
| LL | H2, H4, H10 | H15, H16, H17, H19, H20 | H1 | H1, H4, H6, H7 |
| PL | H11, H12 | H13, H14, H17, H20 | H3, H2 | H2, H9 |
| TB | H5 | H3 | H3 | H5 |
| TC | H1, H5 | H2, H3, H5, H6 | H3, H4, H5 | H2, H8 |
| YA | H1, H2, H4 | H6, H7, H10, H15, H19 | H1, H3, H6 | H2, H6, H7, H8 |
| YH | H4, H15 | H17, H18, H19, H20 | H1 | H4, H9 |

**Table S3. Population clusters of *S. hilpa* found by BAPS**

| Gene | Groups | Populations |
| --- | --- | --- |
| *COI* | HL | HL |
|  | PL | PL |
|  | SS1 | FX, HC, LF, TB, TC, YA |
|  | SS2 | HY, LF, LL, YA |
| *ITS1* | HL | HL |
|  | PL+SS1+SS2 | FX, HC, HY, LF, LL, PL, TB, TC, YA |

Population codes are according to Table 1.

**Table S4. Intraspecific and interspecific genetic distance of *S. yangi* and other related species based on (*COI*+*COII+Cytb+A6A8*) gene**

| Population | FX | TB | TC | HC | YA | PL | LF | YH | LL | HL | *O. canadensis* | *O. utahensis* |
| --- | --- | --- | --- | --- | --- | --- | --- | --- | --- | --- | --- | --- |
| FX | 0.000–0.000 |  |  |  |  |  |  |  |  |  |  |  |
| TB | 0.000–0.001 | 0.000–0.002 |  |  |  |  |  |  |  |  |  |  |
| TC | 0.003–0.003 | 0.003–0.004 | 0.000–0.000 |  |  |  |  |  |  |  |  |  |
| HC | 0.002–0.002 | 0.001–0.002 | 0.001–0.002 | 0.000–0.000 |  |  |  |  |  |  |  |  |
| YA | 0.002–0.005 | 0.002–0.003 | 0.000–0.003 | 0.003–0.005 | 0.000–0.000 |  |  |  |  |  |  |  |
| PL | 0.005–0.006 | 0.007–0.009 | 0.008–0.008 | 0.007–0.009 | 0.006–0.008 | 0.000–0.000 |  |  |  |  |  |  |
| LF | 0.003–0.003 | 0.002–0.003 | 0.002–0.002 | 0.000–0.002 | 0.002–0.006 | 0.007–0.008 | 0.000–0.000 |  |  |  |  |  |
| YH | 0.001–0.003 | 0.001–0.003 | 0.002–0.003 | 0.000–0.003 | 0.001–0.004 | 0.006–0.008 | 0.003–0.003 | 0.000–0.000 |  |  |  |  |
| LL | 0.002–0.002 | 0.002–0.003 | 0.001–0.001 | 0.000–0.001 | 0.005–0.005 | 0.006–0.009 | 0.002–0.002 | 0.005–0.005 | 0.000–0.000 |  |  |  |
| HL | 0.09–0.018 | 0.008–0.015 | 0.009–0.016 | 0.006–0.016 | 0.008–0.012 | 0.008–0.009 | 0.009–0.013 | 0.008–0.011 | 0.008–0.013 | 0.000–0.000 |  |  |
| *O. canadensis* | 0.108–0.110 | 0.118–0.119 | 0.111–0.111 | 0.109–0.110 | 0.109–0.111 | 0.108–0.111 | 0.110–0.110 | 0.109–0.111 | 0.110–0.111 | 0.110–0.110 | 0.000–0.000 |  |
| *O. utahensis* | 0135–0.140 | 0139–0.140 | 0136–0.140 | 0137–0.139 | 0138–0.140 | 0135–0.138 | 0135–0.139 | 0138–0.145 | 0137–0.148 | 0139–0.147 | 0135–0.148 | 0.000–0.000 |

**References**

1. Simon C, et al. Evolution, weighting and phylogenetic utility of mitochondrial gene sequences and a compilation of conserved PCR primers. Ann Entomol Soc Am. 1994;87:651–701.
2. Zahniser JN, Dietrich CH. Phylogeny, evolution, and historical biogeography of the grassland leafhopper tribe Chiasmini (Hemiptera: Cicadellidae: Deltocephalinae). Zool J Linn Soc. 2015;175:473–495.
3. Marshall DC, et al. Steady Plio-Pleistocene diversification and a 2-million-year sympatry threshold in a New Zealand cicada radiation. Mol Phylogenet Evol*.* 2008;48:1054–1066.
4. Arensburger P, et al. Biogeography and phylogeny of the New Zealand cicada genera (Hemiptera: Cicadidae) based on nuclear and mitochondrial DNA data. J Biogeogr. 2004;31:557–569.
5. Ji YJ, Zhang DX, He LJ. Evolutionary conservation and versatility of a new set of primers for amplifying the ribosomal internal transcribed spacer regions in insects and other invertebrates. Mol Ecol Notes. 2003;3:581–585.
