# Supplementary figures and images for: Genetic divergence, population differentiation and phylogeography of the cicada Subpsaltria yangi based on molecular and acoustic data: an example of the early stage of speciation?
Source: BMC Evol Biol. 2019 Jan 8;19:5. doi: 10.1186/s12862-018-1317-8 (PMC6323834; doi:10.1186/s12862-018-1317-8)

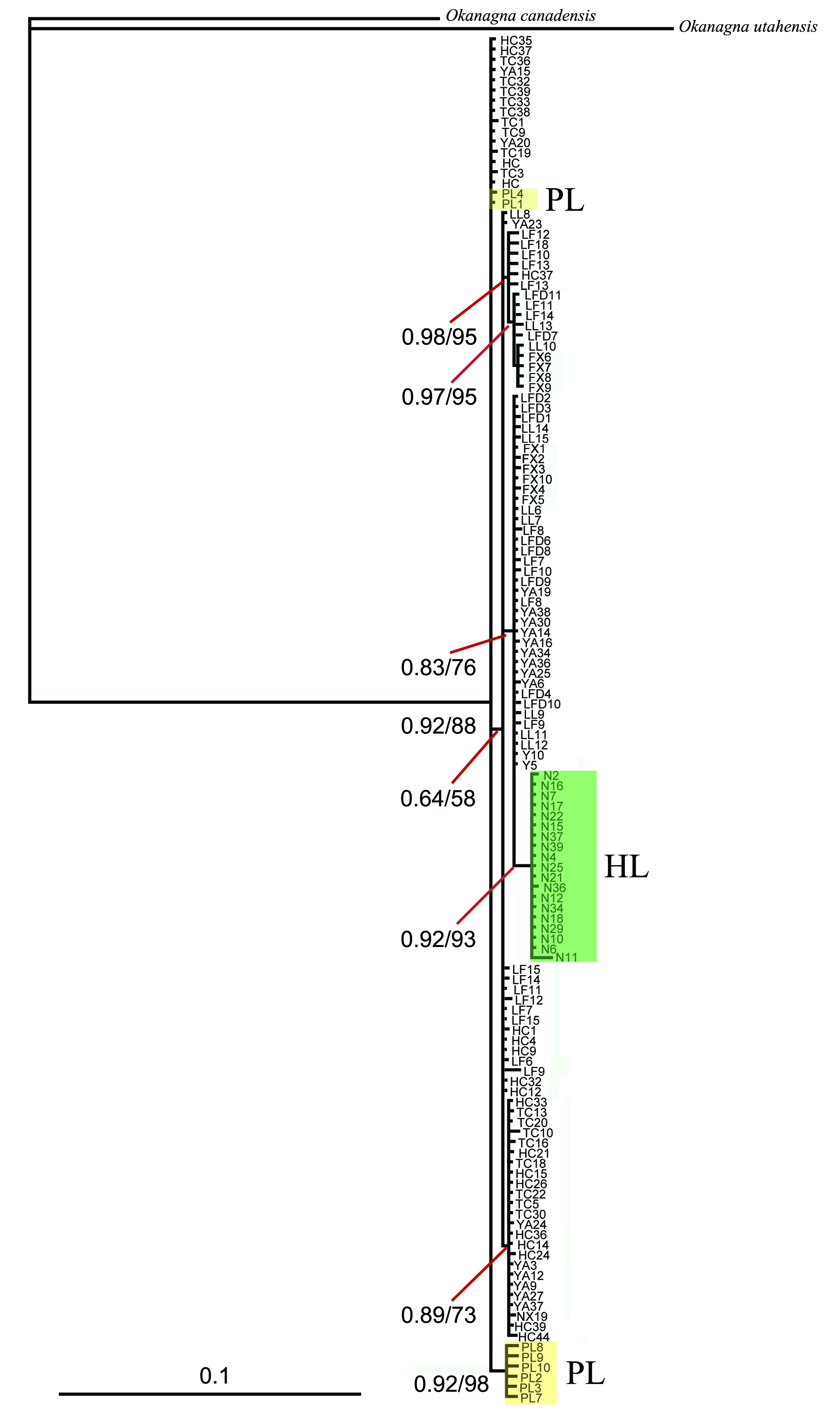

Supplement: Supplementary file 2 — Figure S1. Phylogram reconstructed based (EF-1α + ITS1) genes. Bayesian posterior probabilities and ML bootstrap values are indicated near tree branches. (TIF 1020 kb) [file 12862_2018_1317_MOESM2_ESM.tif]

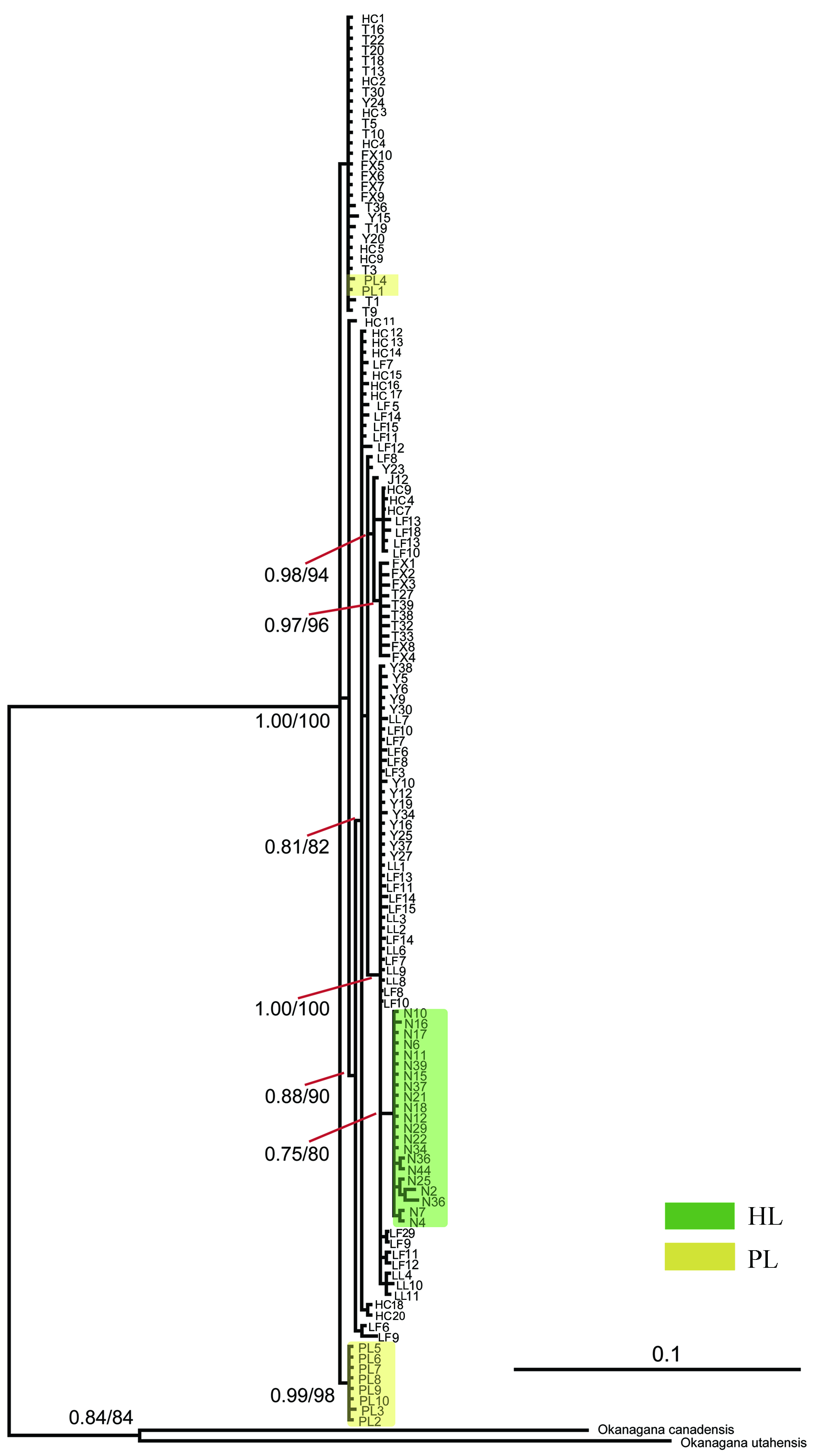

Supplement: Supplementary file 3 — Figure S2. Phylogram reconstructed based (COI + COII + Cytb + A6A8 + EF-1α + ITS1) genes. Bayesian posterior probabilities and ML bootstrap values are indicated near tree branches. (TIF 1513 kb) [file 12862_2018_1317_MOESM3_ESM.tif]

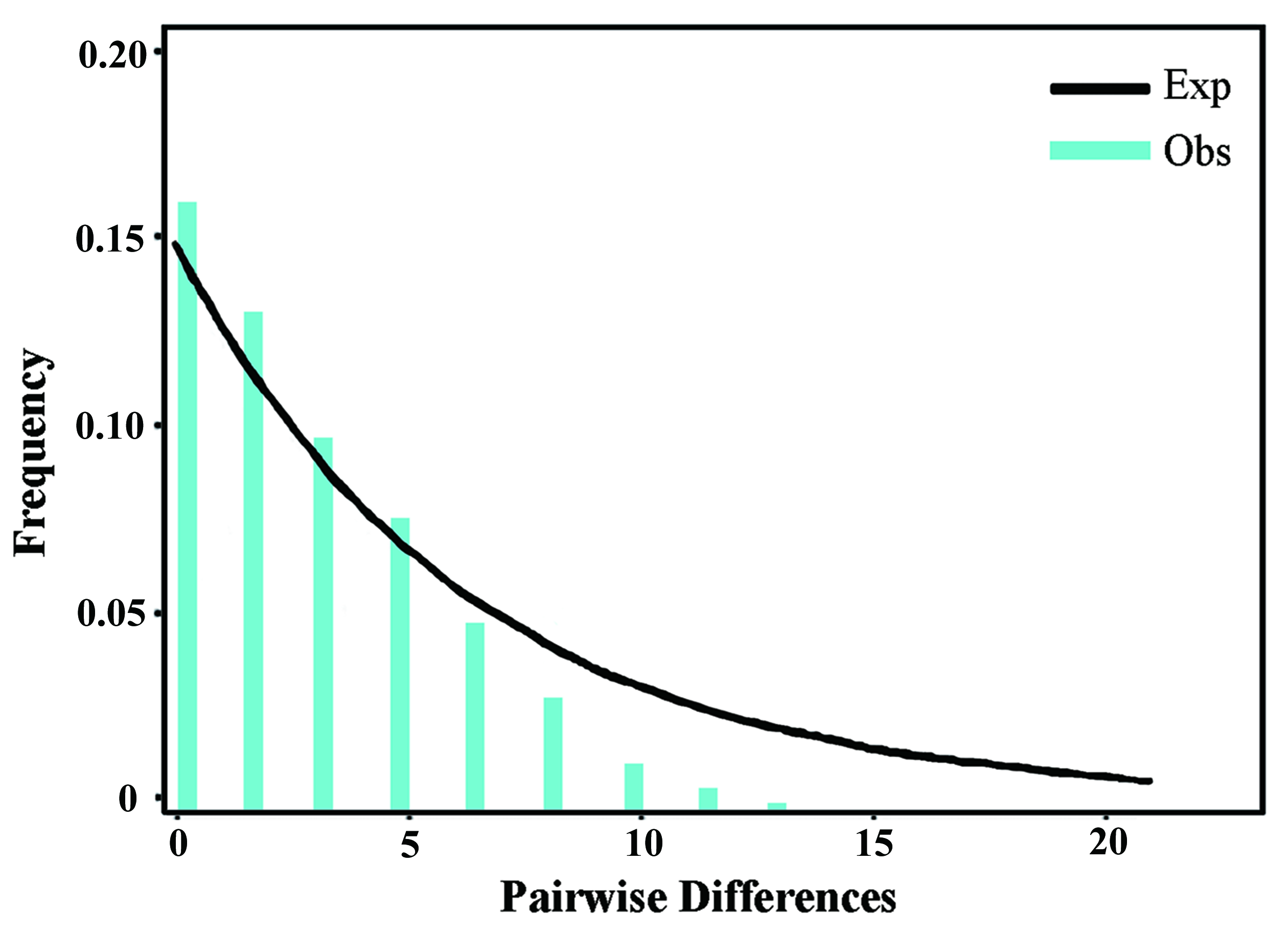

Supplement: Supplementary file 4 — Figure S3. Pairwise mismatch distribution of HL population. X axis: Pairwise Differences. Y axis: Frequency. Obs means the observed distribution of pairwise difference. Exp means the expected equilibrium distributions. (TIF 1187 kb) [file 12862_2018_1317_MOESM4_ESM.tif]

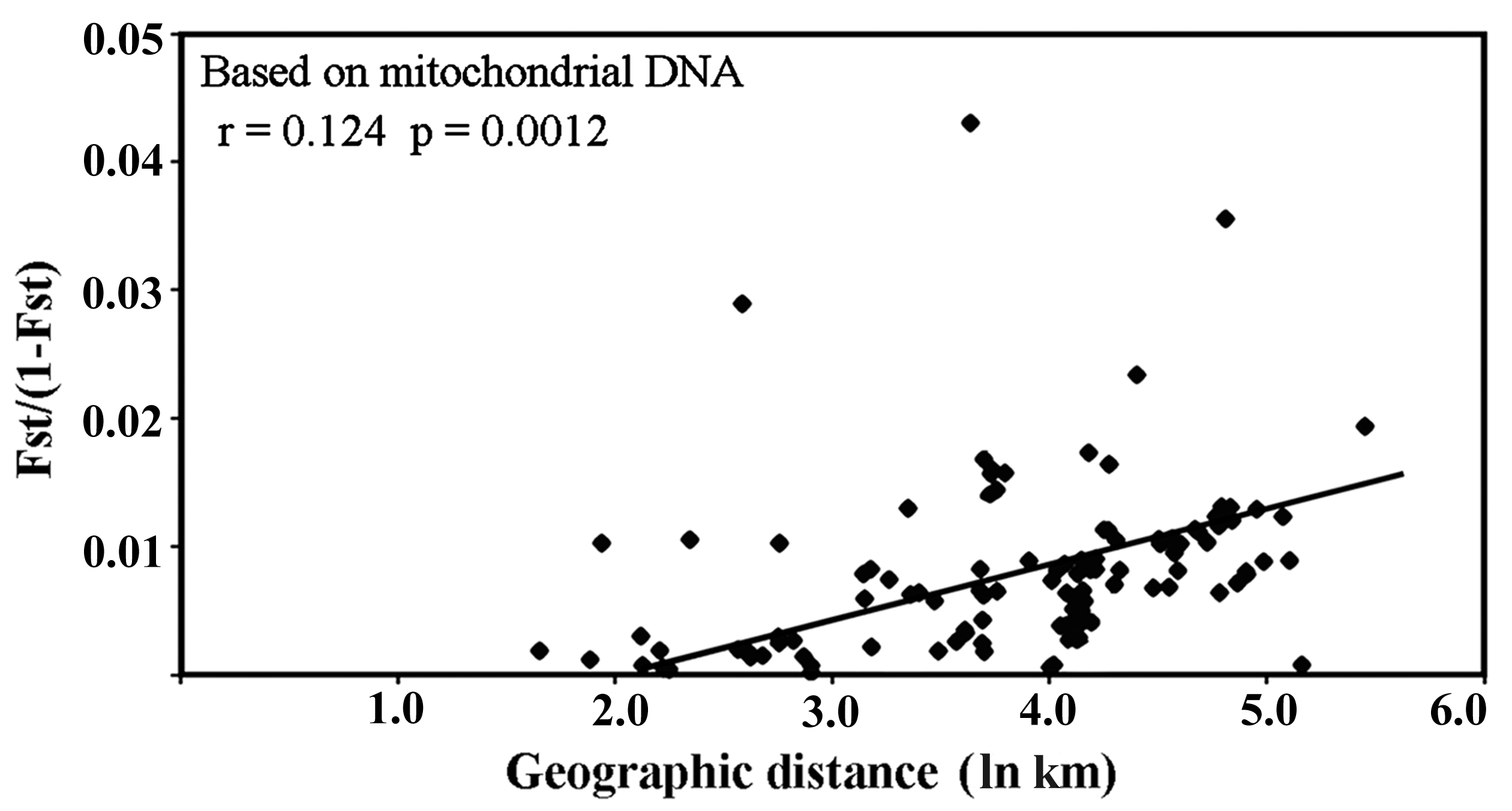

Supplement: Supplementary file 5 — Figure S4. Scatter plots of genetic distance vs. geographical distance for pairwise population comparisons (both analyses are calculated from 100,000 randomizations). (TIF 206 kb) [file 12862_2018_1317_MOESM5_ESM.tif]

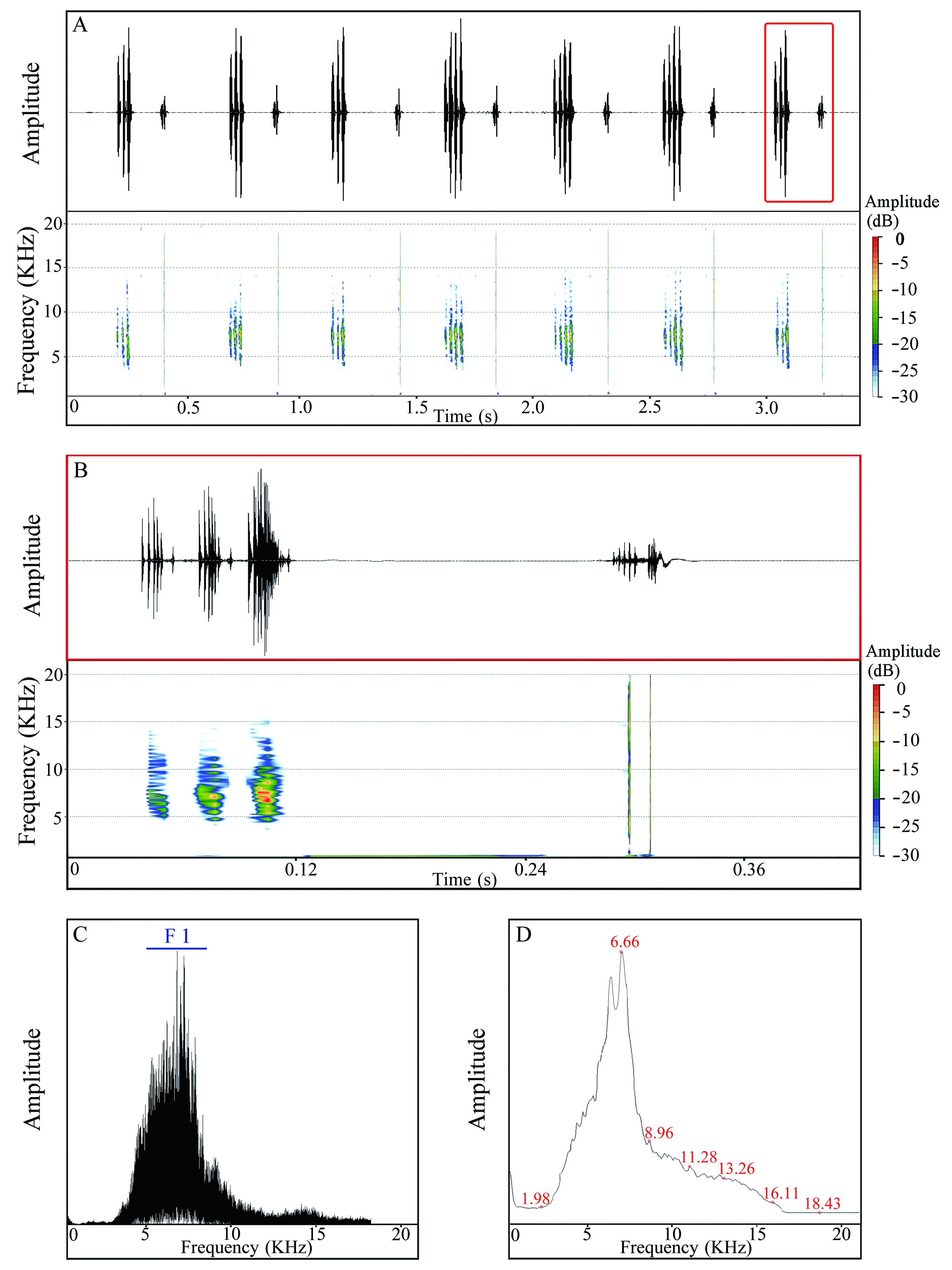

Supplement: Supplementary file 6 — Figure S5. The divergence time analysis of S. yangi based mtDNA gene, using the rate of 2.3% per million years. Estimates of divergence time are shown at nodes above branches. The divergence times occurred at approximately 0.01 Ma and under 0.01 Ma are not shown. (TIF 1010 kb) [file 12862_2018_1317_MOESM6_ESM.tif]

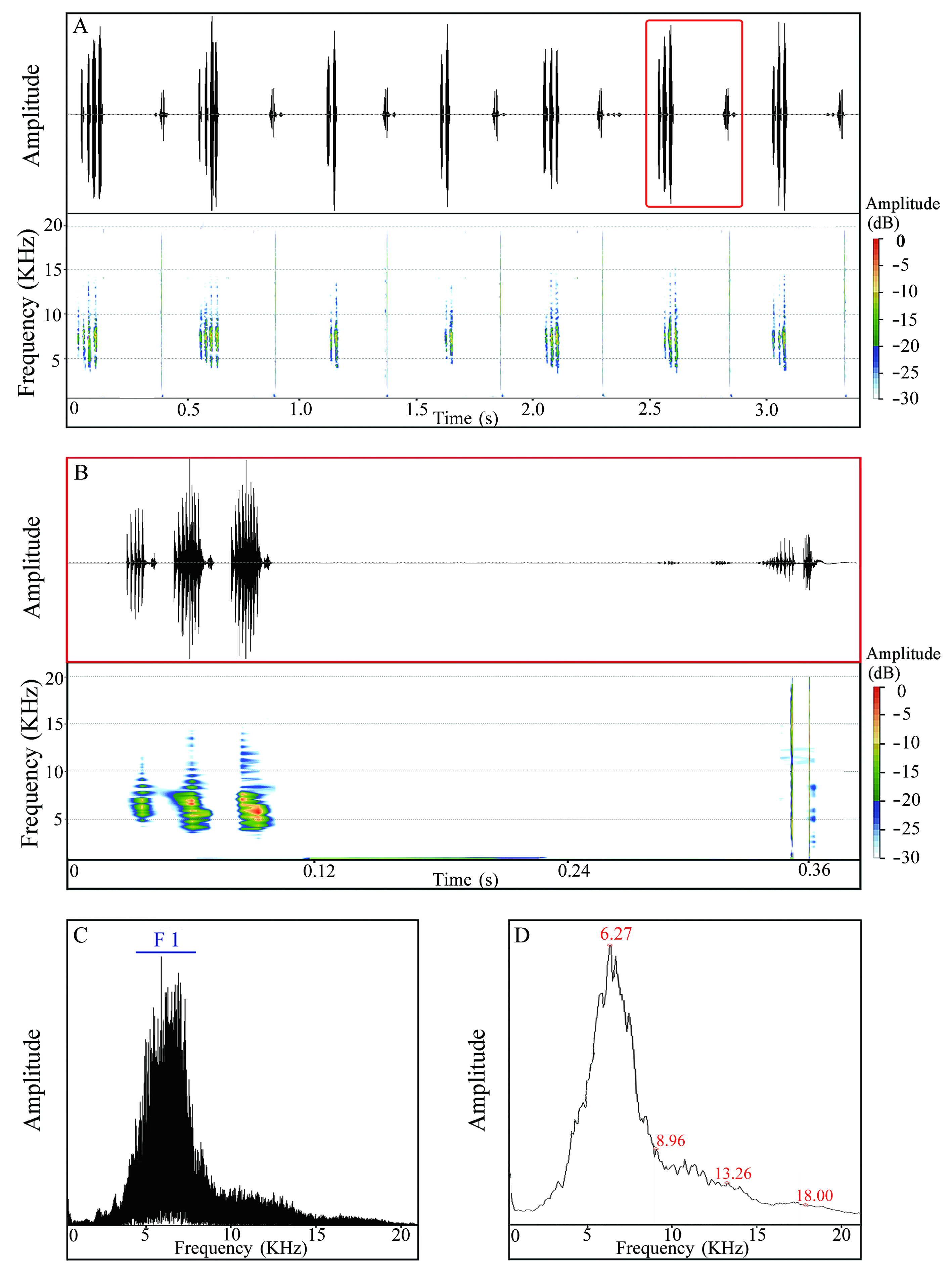

Supplement: Supplementary file 7 — Figure S6. Acoustic analyses of the male calling song structure of S. yangi from Pingliang (PL). A, oscillogram and spectrogram of the timbal and stridulatory sounds were produced alternately (i.e., upward and downward echemes). B, detailed oscillogram and spectrogram of timbal and stridulatory sounds (marked by the red box in A). C, D, power frequency spectrum of the signal showing dominant frequencies marked by F1. (TIF 2804 kb) [file 12862_2018_1317_MOESM7_ESM.tif]

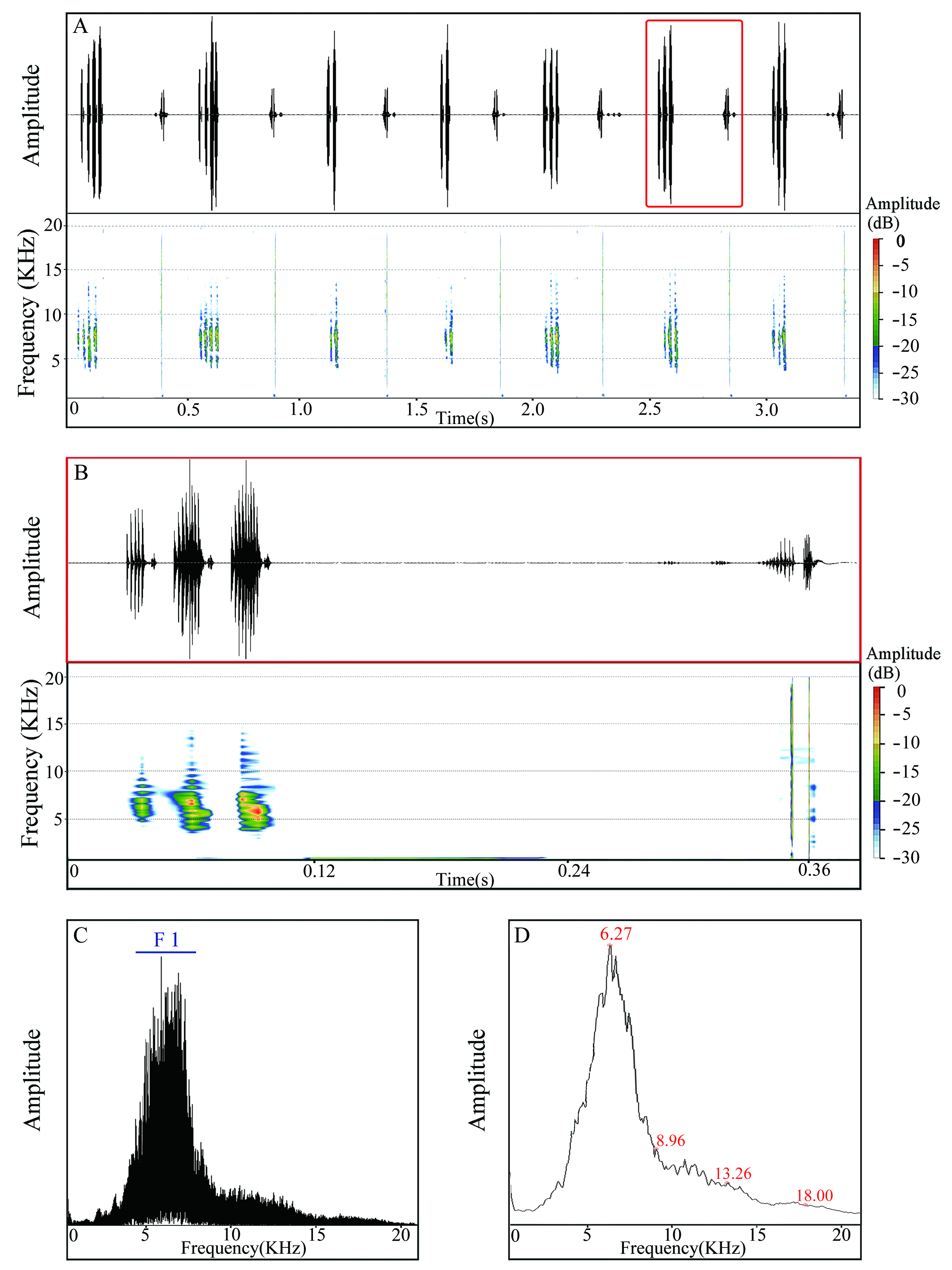

Supplement: Supplementary file 8 — Figure S7. Acoustic analyses of the male calling song structure of S. yangi from Tongchuan (TC). A, oscillogram and spectrogram of the timbal and stridulatory sounds were produced alternately (i.e., upward and downward echemes). B, detailed oscillogram and spectrogram of timbal and stridulatory sounds (marked by the red box in A). C, D, power frequency spectrum of the signal showing dominant frequencies marked by F1. (TIF 3031 kb) [file 12862_2018_1317_MOESM8_ESM.tif]
